# Assessing diagnostic, vaccine and therapeutic potential of selected *Trichinella* proteins^☆^

**DOI:** 10.1016/j.fawpar.2025.e00283

**Published:** 2025-09-01

**Authors:** Anna Stachyra, Justyna Bień-Kalinowska

**Affiliations:** a Department of General Biology and Parasitology, Medical University of Warsaw, Chałubińskiego 5, 02-004 Warsaw, Poland; b Institute of Outcomes Research, Maria Sklodowska-Curie Medical Academy, Warsaw, Pl. Żelaznej Bramy 10, 00-136 Warsaw, Poland

**Keywords:** *Trichinella*, Recombinant proteins, Antigens, Vaccines, Diagnostics, Therapy

## Abstract

Trichinellosis is an important zoonotic parasitosis caused by nematodes of the genus *Trichinella*. In humans, *Trichinella* infection occurs through the ingestion of raw or semi-cooked meat of animals infected with *Trichinella spp.* larvae, as the causative agent. Over the past decade, technological developments have enabled great achievements in the study of the genome, secretome and proteome of *Trichinella*. These achievements provide knowledge to screen, identify, and compare the proteins and antigens involved in the host-parasite communication and interactions with the host's immune system and thus constituting diagnostic, vaccine, or therapeutic targets. Much attention has been focused on identifying and characterizing proteins from different *Trichinella* stages to find molecules useful for serodiagnosis and vaccine development. This review presents a number of recombinant proteins examined as candidates for diagnosis of *Trichinella* infection. However, antigens suitable for improved early diagnosis or detection are not yet available. Identification of potential vaccine candidates against trichinellosis remains a significant challenge. Various recombinant protein vaccines have been reviewed to improve the protective effect against *Trichinella* infection in mice, rat or swine models. A considerable amount of research has investigated the immunomodulatory potential of *Trichinella* proteins. The application of total ES products as well as individual components in recombinant form, showed that they exert strong immunomodulatory effects and can act prophylactically or therapeutically in animal models of autoimmune diseases. This paper provides an overview and summary of recent achievements in the field of *Trichinella* recombinant proteins, emphasizing their potential application to diagnosis, vaccination, and modulation of allergy and oncogenesis.

## Highlights

• Review of *Trichinella* proteins cloned and used in recombinant form.
• Review of three main areas of potential applications of *Trichinella* proteins.
• Review of recombinant vaccines against trichinellosis.
• Review of immunomodulatory properties of recombinant *Trichinella* proteins.

## 1 Introduction

Trichinellosis is a food-borne parasitic zoonosis caused by nematodes of the genus *Trichinella.* These parasitic nematodes are distributed worldwide in domestic and/or wild animals, except in Antarctica, and primarily infect mammals, including humans, but are also known to infect reptiles and predatory birds (Zarlenga et al., 2020). Since infection occurs through the ingestion of meat containing muscle larvae (ML), its natural hosts are carnivores and omnivores. Human trichinellosis is one of the most important parasitic zoonoses, and is caused by consumption of undercooked meat products containing infective larvae (Gottstein et al., 2009). It is included in the European Center for Disease Prevention and Control (ECDC) list as one of eight zoonotic agents subject to compulsory annual monitoring and mandatory reporting of foodborne outbreaks. Reported outbreaks indicate continuous risk of transmission to both domestic animals and humans. The direct source of infection may be pork or game meat - gaining increasing interest among consumers. A vaccine is needed for vaccination of selected high-risk animals, most of all pigs, which is the main host globally, to prevent disease and economic loses.

Besides its public health significance, *Trichinella* is a very interesting research subject and a potential source of clinically useful proteins. *Trichinella* has a unique life cycle among parasitic nematodes, that is confined to a single host. The life cycle includes the molting and maturation of infective larvae in the intestine, followed by the mating of the adult worms (AD), and the subsequent release of newborn larvae (NBL), which migrate to the muscle tissues. After colonization of the striated muscle cells by *Trichinella* larvae, nurse cells develop within 15–20 days, enabling the larvae to remain metabolically active until they are ingested by the next potential host (Bruschi and J, 2014). In these colonization processes the excretory-secreted (ES) products of the parasites, originated from both AD and ML stages, are thought to drive invasion, migration, or the remodeling of host cells and tissues which support parasite development. ES products are a mixture of secreted proteins including proteases, protease inhibitors, allergen homologs, glycolytic enzymes, lipids, glycans, and extracellular vesicles. They take part in communicating with the host at the molecular level (Hewitson et al., 2009). The majority of ES proteins are glycoproteins, some of them bearing multi-antennary *N*-glycans capped with tyvelose (Denkers et al., 1990). Tyvelose creates a unique epitope on *T. spiralis* glycan antigens of ML, which provokes synthesis of parasite-specific antibodies during the muscle stage of the infection. Such antibodies provide protection from reinfection and, when applied *in vitro*, inhibit migration into the cell layer and affect molting of invading muscle larvae (McVay et al., 1998).

*T. spiralis* modulates and downregulates the host's immune response by inducing regulatory T cells, tolerogenic dendritic cells, and the production of anti-inflammatory and regulatory cytokines (Ilic et al., 2012). Other parasitic nematode infections are usually associated with Th2-type immune responses, but during the intestinal phase of infection, *Trichinella* benefits from the strong Th1-type immune response, that controls the magnitude of newborn larvae release and prevents massive muscle invasion and potential host death. After several days AD are expelled from the intestine and NBL reach the muscle cells initiating the muscle phase. This is the moment when a mixed Th1/Th2 response is induced, with predomination of Th2, and strong activation of regulatory mechanisms. During this phase regulatory response and immunological homeostasis provides the survival of both parasite and the host, as *Trichinella* occupies the host's muscle cells without killing them. *Trichinella* is a potent immunomodulator and proteins involved in the process of immunomodulation are potential targets for clinical application.

**Fig. 1:**
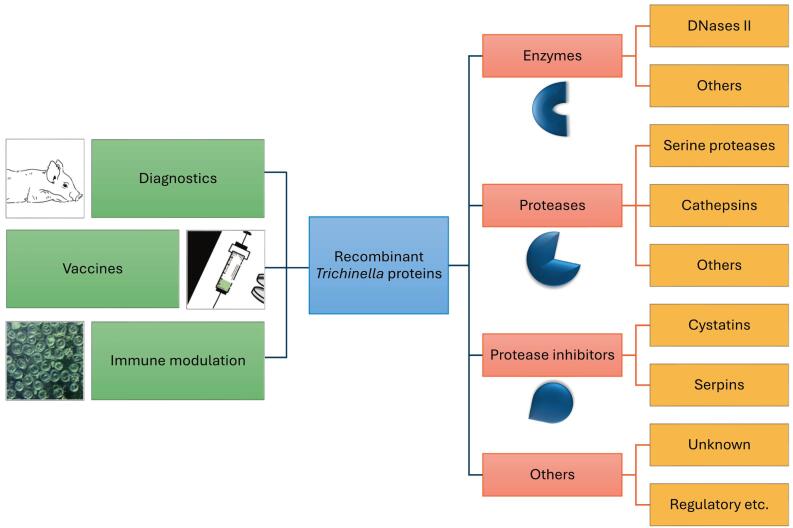
Groups of recombinant *Trichinella* proteins and their potential application.

Many recent studies have investigated *Trichinella* proteins, their functions in the complex life cycle and their potential application in diagnosing trichinellosis, developing vaccines against infection, and treating autoimmune and inflammatory diseases (Fig. 1). This review emphasizes publications from the last 10 years (2015–2024), but references older publications as necessary. During this period, acceleration in research concerning identification and cloning of *Trichinella* proteins has taken place and experiments have been conducted to develop new diagnostic tests (Gómez-Morales et al., 2022), anti-*Trichinella* vaccines (Tang et al., 2022) and new immunomodulatory molecules (Bohnacker et al., 2020). Especially, the last goal seems to be gaining increasing attention. We group publications concerning cloning, production and use of recombinant *Trichinella* proteins into the following categories: (a) enzymes involved in basic cellular processes; (b) proteases; (c) protease inhibitors; (d) other proteins of less clear function.

To date about 80 *Trichinella* proteins have been subjected to molecular cloning. They were most often identified during immunoblot-based proteomic analysis or immuno-screening of cDNA expression libraries, and chosen based on their putative roles in parasite infection processor suspected immunomodulatory potential, as well as insights gained from studies on other species of parasites. Only a limited number of proteins were examined so far, *e.g.* as vaccine candidates or immunomodulators (Bruschi et al., 2022). Therefore, selecting *Trichinella* proteins that may play a key role in communication with the host and interact with the host's immune system and serve as diagnostics, vaccine or therapeutic targets, is the right direction for future study.

## 2 Enzymes involved in basic cellular processes

With the development of genomics, proteomics, and transcriptomics, proteins of *Trichinella* were isolated and identified. Many different *Trichinella* proteins involved in host-parasite interaction that have been cloned and used in recombinant form are enzymes. They are involved in crucial roles during parasite development and infection, and include housekeeping enzymes responsible for basic cellular processes, proteins folding and modification, perforating host tissues, affecting host metabolic pathways, as well as interacting with host immune cells to modulate and downregulate the immune response. To be able to perform diversified functions, some enzymes are likely multifunctional proteins or moonlighting proteins (Jeffery, 2018). They acquired non-enzymatic function and serve also as receptors, secreted cytokines, transcription factors, *etc.* The function of a moonlighting protein can vary depending on its location within the cell and/or the type of the cell in which it is expressed. Large subgroups of moonlighting proteins are intracellular enzymes that perform a secondary function when secreted outside the cell or attached to the cell surface (Copley, 2003). These features align well with parasites like *Trichinella*, as parasites proteins are intended to interact with the host. Proteins belonging to this group may serve as important targets for vaccines and immunomodulation. Enzymes that were used are listed in Table 1.

**Table 1:** Recombinant *Trichinella* enzymes.

Protein group	Name in the reference	Unified name	Accession number	Applicational context	ML reduction*	Reference
DNase II-like	TsDNase II	DNase II	AAY32316.1^a^	Vaccines	52–59 %	(Qi et al., 2018b; Qi et al., 2018a)
	Ts87 protein	DNase II	AAK85403.1^a^	Vaccines	43 %	(Yang et al., 2013)
	DNase II-7	DNase II	AAY32322.1	Vaccines	45 %	(Xu et al., 2021b; Qi et al., 2018b)
	SS1	DNase II	AAK16519.1	Vaccines	67 %	(Xue et al., 2021a)
	43-kDa protein	DNase II	AAA30327.1	Vaccines, Immune modulation	45 %	(Wang et al., 2021a; Wang et al., 2018; Wang et al., 2021b; Ma et al., 2024)
	Plancitoxin-1	DNase II	XP_003370763.1	Vaccines	40 %	(Ding et al., 2024)
Other enzymes	Ts-FBPA	Fructose-1,6-bisphosphate Aldolase	XP_003374282.1	Vaccines	52 %	(Yang et al., 2019)
	TsPPase	Inorganic pyrophosphatase	XP_003371891.1	Vaccines	51 %	(Hu et al., 2021)
	TsNd	Nudix hydrolase	ABY60748.1	Diagnostics, Vaccines	49–57 %	(Wang et al., 2021a; Long et al., 2015; Long et al., 2014; Liu et al., 2015a; Liu et al., 2015b)
	Ts-SUCLA-β	Succinate Coenzyme A Ligase B	KRY28749.1	Vaccines, Immune modulation	20 %	(Aimulajiang et al., 2024; Sun et al., 2019c)
	TsTPX2	Thioredoxin Peroxidase 2	KRY40252.1	Immune modulation	na	(Jin et al., 2020a)
	TsGST	Glutathione-S-transferase	XP_003371755.1	Vaccines	38 %	(Li et al., 2015)
	TspGST	Glutathione-S-transferase	XP_003373651.1	Vaccines	43 %	(Liu et al., 2017)
	TsENO	Enolase	AAK50056.1	Vaccines	17 %	(Zhang et al., 2018b)

* - reduction after single protein immunization, combination of two proteins is not shown; ^a^ – sequences shares 95.29 % identity;

### 2.1 DNase II

DNase II mainly exists in lysosomes and nuclei, and plays an important role in pathogen invasion and evasion of the immune response of the host (Evans and Aguilera, 2003). Among enzymes, DNases II are one of the most studied *Trichinella* enzymes (Wang et al., 2023). It is assumed that DNase II plays a crucial role during the intestinal phase of invasion. Its immunomodulatory properties, by influencing host immune cells and protecting NBL from innate immunity, have been experimentally confirmed. The protective potential of DNase II was established *via* immunization experiments in mice. The only study to date using pigs was conducted by Xu et al. (2021b). Vaccination induced a mixed Th1/Th2 immune response with a predominance of Th1 cytokines IFN-γ, IL-2 in experimental pigs, as well as significant increases in IgG, IgG1, IgG2a, and IgM. Vaccinated pigs exhibited a 46 % reduction in ML burden compared to the control group and histological analysis showed altered morphology of cysts and reduced inflammatory cell infiltration around cysts in vaccinated pigs. There are very few other publications concerning anti-*Trichinella* vaccination using pigs, the most important *Trichinella* host, from the standpoint of food safety.

Another study characterized two adult-specific DNase II proteins (TsDNase II-1 and TsDNase II-7) and evaluated their potential as vaccine targets in mouse immunization experiments (Qi et al., 2018b). Immunization of mice with TsDNase II-1, TsDNase II-7 or a protein combination generated a significant humoral immune response. IgG1 levels were higher than IgG2a, indicating a Th2-predominant response. AD reduction was 40 % for TsDNase II-1, 35 % for TsDNase II-7 and 50 % for combination, while ML reduction was 50 % for TsDNase II-1, 42 % for TsDNase II-7 and 58 % for combination. DNase II-1 was more effective in eliciting an immune response and protection than DNase II-7, but the combination of both proteins provided broader and stronger immunity.

Another immunization study with a combination of proteins used DNase and protease and they were used in the form of a DNA vaccine (Wang et al., 2018). Mice immunized with the plasmid constructs pVAX1-Ts43 (DNase) and pVAX1-Ts45 (protease) elicited 52 % and 34 % reductions in ML. The combined immunity of two constructs induced better immune responses and provided 76 % reductions in ML.

A different combination strategy used DNA and protein DNase II vaccine alone or together for immunization of mice. Co-immunization with both formulations reduced ML burden by 44 %, while protein-only immunization reduced burden by 40 %, and DNA-only immunization achieved 10 % reduction. Co-immunized and protein-immunized mice exhibited higher total IgG levels than DNA-only mice. IgG2a/IgG1 ratios indicated a balanced Th1/Th2 response in all groups. Co-immunization significantly elevated IL-2, IL-4, IL-6, and IFN-γ levels compared to single immunizations, indicating mixed Th1/Th2 responses and relatively suppressed IL-17 and IL-10 levels. Additionally co-immunized mice showed significantly enhanced T cell proliferation upon stimulation (Yang et al., 2013).

In several studies, DNase II DNA vaccine was orally delivered by bacterial vectors. Two of these studies used attenuated *Salmonella.* In the first study, vaccinated mice showed a 54 % reduction in AD burden and a 60 % reduction in ML. Also, female fecundity was significantly reduced (Qi et al., 2018a). In the second study vaccinated mice exhibited a 46 % reduction in ML burden (Wang et al., 2021b). In both studies significant production of specific IgG and intestinal secretory IgA antibodies were detected, associated with increased cytokine secretion (IFN-γ, IL-4, IL-10, IL-17 A). Two other studies used *Lactobacillus*. In the first study, vaccinated mice showed a 46 % reduction in AD burden and a 67 % reduction in ML (Xue et al., 2021a). In the second study DNAse II was used in combination with another enzyme, nudix hydrolase. Vaccinated mice showed a 76 % reduction in AD and a 57 % reduction in ML (Wang et al., 2021a). Specific IgG, Th1/Th2 cytokines, and secreted IgA were also detected.

The study of Ding et al. (2024) investigated the potential of DNase II as a vaccine candidate in mice, but recombinant protein was produced in a Chinese hamster ovary (CHO) cell mammalian expression system. Specific antibodies in the sera of immunized mice were confirmed but animals showed only a 28 % reduction in AD and a 40 % reduction in ML. Several cell culture experiments were conducted to investigate how *T. spiralis* interacts with host immune cells, focusing on extracellular trap (ET) formation and parasite evasion mechanisms. It was found that *T. spiralis* ES degrades ETs from neutrophils and macrophages, presumably due to nuclease activity of DNase II.

Only one study to date investigated DNase II immunomodulatory potential *in vivo* (Ma et al., 2024). It explored how DNase induces indoleamine 2,3-dioxygenase (IDO) expression, regulates CD4+ T cells and reduces inflammation and tissue damage in a mouse rheumatoid arthritis (RA) model. DNase significantly increased IDO expression in cultured dendritic cells (DCs) compared to the control, suppressed CD4+ T cell proliferation and increased their apoptosis *via* the IDO pathway. This was evidenced by higher levels of pro-apoptotic proteins. In the *in vivo* experiment it reduced cartilage and bone damage, caused minimal inflammatory infiltration and less edema in ankle joints of treated mice. The authors proposed that IDO depleted tryptophan in the local microenvironment, leading to impaired T cell function, inhibited proliferation, and increased apoptosis. These effects alleviated RA symptoms by reducing immune overactivation.

These studies have indicated that the DNase II can provide a high rate of protection against *T. spiralis* infection (up to 76 %) when is used in the form of a DNA vaccine: delivered by a bacterial vector (*Lactobacillus*) or as a combination of DNase with another protein construct. As DNase plays important roles during the intestinal phase, inducing a local intestine response by oral delivery was an effective strategy. Future research should include testing different combinations of DNase with other antigens, since this strategy has proved promising. The potential immunomodulatory effects of DNase are promising, as shown by the idea for treatment of RA, and research should be continued.

### 2.2 Other enzymes

A number of other enzymes have been cloned and used in experiments. Mostly these are single publications and continuation studies are yet to come. Several publications focus on cloning and functional analysis of the enzymes, without pursuing their potential application. Those publications are not included in this review, but merit follow up. Nudix hydrolase (Nd) stands out in this context and was investigated the most. It hydrolyze nucleoside diphosphate derivatives to release nucleoside monophosphates and other by-products. This protein is expressed by all developmental stages of *T. spiralis* and it was shown to bind to intestinal epithelial cells (IECs), playing a role in mediating invasion.

The aim of the first study was to evaluate the potential of recombinant nudix hydrolase protein from *T. spiralis* as a serodiagnostic antigen for detecting trichinellosis using enzyme linked immunosorbent assay (ELISA) (Long et al., 2015). rTsNd-ELISA demonstrated 100 % sensitivity and specificity in sera of mice infected with various *Trichinella* species and other parasites, making it a promising candidate for serodiagnosis of trichinellosis. However, it showed slightly delayed antibody detection compared to ES-ELISA and lower detection rates for non-encapsulated *Trichinella* species, such as *T. pseudospiralis*.

Several studies were conducted to evaluate Nd potential as a vaccine candidate to induce protective immunity against trichinellosis in mice. In a preliminary study subcutaneously immunized mice showed a strong humoral immune response with higher IgG1 than IgG2a and vaccination led to a 58 % reduction in AD and a 57 % reduction in ML compared to controls (Long et al., 2014). The study of Wang et al. (2021a) used Nd in combination with DNase as DNA in a *Lactobacillus* vector. Oraly vaccinated mice showed a 76 % reduction in AD and 57 % reduction in ML. In another study Nd DNA vaccine was injected intramuscularly. Vaccinated mice showed significantly elevated serum IgG, with a Th2-skewed response (higher IgG1 than IgG2a), increased intestinal IgA levels and increased levels of both Th1 cytokines (IFN-γ, IL-2) and Th2 cytokines (IL-4, IL-10). Mice showed a 40 % reduction in AD and a 54 % reduction in ML burden compared to controls (Liu et al., 2015a).

In a related study mice were orally vaccinated with Nd DNA delivered by attenuated *Salmonella.* Vaccinated mice showed a significant increase in local mucosal secretory IgA in intestinal washings as well as total IgG levels, with IgG1 being more dominant than IgG2a. Significant reduction in parasite burden was observed, 73 % reduction in AD burden in intestines and 50 % reduction in ML burden. Female adult worms recovered from vaccinated mice were shorter than those from the control group, suggesting reduced fecundity and infectivity (Liu et al., 2015b).

Another enzyme is succinate coenzyme A ligase β-like protein (SUCLA-β), one of the *T. spiralis* ML ES products that plays a role in the citric acid cycle, but may also play an immunomodulatory role as a moonlighting protein. Its vaccine and immunomodulatory potential was studied (Sun et al., 2019c). The experiment was conducted on rats, an important *Trichinella* host. Immunization significantly suppressed IL-17 secretion *in vivo* for the first five days, but no significant changes were observed in other cytokines (IFN-γ, IL-4, IL-9, and TGF-β). The immunized group showed significantly increased levels of IgG, IgG1, and IgG2a antibodies starting from day 21. No significant reduction in AD was observed, but a 21 % reduction in ML burden was noted. Additionally, rat peripheral blood mononuclear cells (PMBCs) were treated with SUCLA-β *in vitro* and it was shown that it suppressed PBMC functions, including migration, proliferation and phagocytosis and also specifically bound to PBMCs.

Another study examined the therapeutic potential of SUCLA-β in managing ovalbumin (OVA)-induced allergic asthma in mice (Aimulajiang et al., 2024). Treatment with rTs-SUCLA-β significantly decreased eosinophil infiltration and inflammation in the lungs. It also reduced eosinophil, neutrophil, lymphocyte, and macrophage counts in bronchoalveolar lavage fluid (BALF), as well as levels of pro-inflammatory cytokines IL-9, IL-17 A, and IFN-γ. It shifted the immune response from pro-inflammatory (Th17) to regulatory (Treg), increased anti-inflammatory cytokine IL-10 and reduced OVA-specific IgE levels. Preventive and therapeutic treatments with 50 μg of rTs-SUCLA-β achieved the most effective reduction.

Another enzyme used in immunization studies was fructose-1,6-bisphosphate aldolase (FBPA) from *T. spiralis* (Yang et al., 2019). It is a key enzyme in glycolysis: a class-I aldolase with conserved catalytic and actin-binding residues. It probably also plays non-glycolytic roles, including host interaction. Mice immunized with rTs-FBPA showed a 49 % reduction in AD and a 53 % reduction in ML burden. The vaccine elicited a mixed Th1/Th2 immune response, with increased levels of specific IgG, IgG1, IgG2a, and IgE antibodies. Splenocytes from vaccinated mice produced elevated levels of cytokines IL-2, IFN-γ, IL-4, and IL-10, indicating robust cellular immunity.

Another glycolytic enzyme used in immunization studies is enolase. It is found on the cell surface of some parasites, aiding in host infection and immune evasion as a moonlighting protein. Vaccination of mice with recombinant enolase protein or a DNA vaccine encoding enolase induced mixed Th1/Th2 responses, but reduction in ML burden was 18 % in the recombinant protein group and 16 % in the DNA vaccine group (Zhang et al., 2018b).

The enzyme inorganic pyrophosphatase, played a critical role in *T. spiralis* larval molting and development (Hu et al., 2021). The study focused on developing an effective oral DNA vaccine delivered with *Lactobacillus*. Mice vaccinated with recombinant *L. plantarum* exhibited significant increases in IgG, IgG1, and IgG2a antibodies and mucosal secretory IgA in the intestines. Cytokine analysis revealed a mixed Th1/Th2 immune response, with increased levels of IFN-γ and IL-4. After challenge with *T. spiralis*, vaccinated mice showed a 55 % reduction in AD and a 52 % reduction in ML.

The last studies with enzyme immunization reviewed here, used glutathione-S-transferase (GST), a detoxification enzyme important for parasite survival. Li et al. (2015) showed that mice vaccinated with rTsGST developed a Th2 predominant immune response. Mice immunized with rTsGST showed only 36 % reduction in AD burden and 39 % reduction in ML. In a second study GST of a different sequence was used. Vaccinated mice also showed a Th2-dominant response. Protective immunity detected was 34 % reduction in AD and 44 % reduction in ML burden (Liu et al., 2017).

An enzyme used in an immunomodulation study was thioredoxin peroxidase 2 (TPX2), belonging to the family of antioxidant enzymes characterized by 2-cys residues, which protect helminths from host reactive oxygen species (ROS) (Jin et al., 2020a). rTsTPX2 directly drove mice peritoneal macrophages and RAW264.7 cell line to polarize into the M2 phenotype *in vitro*. These M2 macrophages promoted a Th2-skewed immune response, highlighting their role in protective immunity after adoptive transfer. In this study, mouse immunizations were also conducted. The levels of Th2 cytokines IL-4 and IL-10 were significantly higher in immunized mice, while Th1 cytokines, IFN-γ and TNF-α, were significantly suppressed. Flow cytometry analysis revealed a Th2-skewed immune response.

The studies reviewed here showed that apart from Nd none of the enzymes assured a high level of protection. Interestingly, when Nd was delivered in bacterial vectors a high reduction of AD burden was observed (>70 %), but ML reduction was significantly lower. This phenomenon needs further investigation, but it may be related to enzymatic activity of the recombinant Nd. This activity could affect vaccine efficacy by regulating the host immune system, after the initial success of the local gut response and the limitation of the formation of AD. Parasite enzymes have both regulatory and antigenic properties for the host immune system. Distinguishing the immune regulatory effects by weakening their activity without changing protein structure may be a good direction for future vaccine research. The application of potential immunomodulatory properties against allergic asthma was studied for SUCLA-β and it is worth further research.

## 3 Proteases

Among the enzymes, proteases warrant a separate category as they have been cloned and used in research in recombinant form at least as frequently as all other enzymes together. Importantly, amino acid sequences of identified proteases usually don't share much similarity (Supplementary file). They are listed in Table 2 and those which share more similarity are marked. Proteases play a key role in larval intrusion, molting and development of the worm. Many studies affirm their role in attachment and entry into IECs. At the same time, they are often involved in interacting with immune cells.

**Table 2:** Recombinant *Trichinella* proteases.

Protein group	Name in the reference	Unified name	Accession number	Applicational context	ML reduction*	Reference
Serine proteases	Ts-NBLsp NBL-SP	Serine protease	AAR36900.1	Vaccines, Immune modulation	77 %	(Xu et al., 2017a; Qu et al., 2020; Long et al., 2021)
	Ts-ADsp-7 Ts-Adsp TsSP Ts-ADpsp	Serine protease	ABY60762.1	Diagnostics, Vaccines, Immune modulation	50–69 %	(Sun et al., 2018a; Sun et al., 2019b; Sun et al., 2019a; Sun et al., 2018b; Gao et al., 2018; Pang et al., 2020; Xu et al., 2020a; Xu et al., 2021a; Xue et al., 2022)
	TsSP1.1	Serine protease	ACA28930.1	Vaccines	42 %	(Bai et al., 2022)
	TsSerp	Serine protease	AAK31787.1^a^	Vaccines	52 %	(Yue et al., 2020)
	TspSP-1.3	Serine protease	ACA28932.1^a^	Vaccines	39 %	(Li et al., 2013)
	TsSP1.2	Serine protease	ACA28931.1	Vaccines	71 %	(Li et al., 2018)
	TbCTRL	Chymotripsin like protease	UPO81526.1^b^	Diagnostics	na	(Grzelak et al., 2022)
	Ts31 31 kDa protein	Chymotripsin like protease	AAA20539.1^b^	Diagnostics, Vaccines	53 %	(Cui et al., 2015; Wang et al., 2015; Ren et al., 2018; Wang et al., 2018)
	TsEla TsE	Elastase	EFV56917.1	Diagnostics, Vaccines	64–69 %	(Hu et al., 2020; Hu et al., 2021; Zhang et al., 2020a; Zhang et al., 2022)
	TpaPLAT	Tissue-type plasminogen activator	KRZ67526.1	Diagnostics	na	(Sahaisook et al., 2022)
	TsP	Peptidase	XP_003379348.1	Vaccines	41 %	(Lei et al., 2020)
	TsT	Trypsin	XP_003374437.1	Vaccines	37 %	(Zhang et al., 2020b)
	TsTryp	Trypsin	XP_003381667.1	Detection	na	(Han et al., 2024)
Cathepsins	TsCPB2	Cathepsin B	ARG41672.1	Vaccines	51 %	(Yang et al., 2018)
	TsCB	Cathepsin B	XP_003379650.1	Vaccines	50 %	(Cui et al., 2019)
	TsCPB	Cathepsin B	AGR34128.1	Immune modulation	na	(Liu et al., 2015c)
	TsCPF1	Cathepsin F	XP_003378245.1	Vaccines	58 %	(Xue et al., 2021b)
	TsCatL2	Cathepsin L	KRY31298.1	Immune modulation	na	(Liu et al., 2023)
	TsCX	Cathepsin X	XP_003372938.1	Vaccines	50 %	(Zeng et al., 2021)
	TsDPP1	Dipeptidyl peptidase	XP_003379334.1	Immune modulation	na	(Yan et al., 2023)
	TsCP	Cysteine protease	XP_003377082.1	Vaccines	33 %	(Song et al., 2018b)
Other proteases	TsAP	Aminopeptidase	EFV57052.1	Vaccines	59 %	(Zhang et al., 2013)
	TsAPP	Aminopeptidase	EFV57850.1	Vaccines	50 %	(Zeng et al., 2021)
	TsASP2	Aspartyl protease	XP_003380300.1	Vaccines	55 %	(Xu et al., 2021c)
	TsASP1	Aspartyl protease	QOQ72494.1	Vaccines	61 %	(Xu et al., 2021d)

* - reduction after single protein immunization, combination of two proteins is not shown; ^a^ – sequences shares 98.81 % identity; ^b^ – sequences shares 96.95 % identity;

### 3.1 Serine proteases

Serine proteases are the most common proteases in *Trichinella* and are also very diverse. They have been used in experiments for serodiagnosis, immunization studies and immunomodulation studies. The first study focused on chymotrypsin (CTR)-like protease, named as 31 kDa protein, and its potential for improving serodiagnosis of trichinellosis (Cui et al., 2015). CTR was expressed in an *E. coli* system. It was recognized in all developmental stages of the parasite and localized to the cuticle and stichocytes. The rTs31-based ELISA demonstrated higher sensitivity (98 %) and specificity (99 %) than the conventional ES antigen ELISA for detecting anti-*Trichinella* IgG antibodies. It exhibited minimal cross-reactivity with sera from patients with other parasitic diseases.

A related study used sera from experimentally infected mice (Wang et al., 2015). ELISA with recombinant antigen and with ES antigens showed no significant difference in performance. Recombinant antigen ELISA exhibited low cross-reactivity with other parasitic infections and performed well for encapsulated *Trichinella* species, but not for non-encapsulated species. In a continuation study, immune response analysis to CTR immunization of mice was performed, as well as protein interaction assays (Ren et al., 2018). rTs31 specifically bound to IECs, entering the cytoplasm and played a role in larval invasion. Vaccination with rTs31 reduced AD by 57 % and ML by 54 %, demonstrating its potential as a vaccine target. Pre-incubation with anti-rTs31 serum significantly reduced larval invasion rates compared to controls as antibodies inhibited larval invasion of IECs and mediated killing of NBL *via* ADCC.

The diagnostic potential of *T. britovi* CTR for detecting IgG antibodies in serum samples from mice and pigs experimentally infected with *T. britovi* or *T. spiralis* was evaluated. The protein was cloned and expressed in *Pichia pastoris*. The study revealed that rTbCTR was a sensitive protein for ELISA serodiagnosis. Anti-CTR IgG levels increased at 41 days post-infection (dpi) for *T. britovi* and 45 dpi for *T. spiralis* in pig sera, indicating that rTbCTR had diagnostic potential for trichinellosis, with varying sensitivity and specificity (Grzelak et al., 2022).

The published results demonstrate that CTR facilitates larval intrusion of the host's enteral epithelium and could be a candidate for a vaccine against the enteral invasive phase of *Trichinella.* In the previously discussed study, CTR was used in the form of a DNA vaccine in combination with DNase II (Wang et al., 2018). Immunized mice elicited partial protective immunity against challenge infections with *T. spiralis* as shown by 34 % reduction in ML with CTR alone. However, the combined immunity of the two constructs induced better immune responses and provided 76 % reduction in ML.

The most frequently studied serine protease is one initially identified in the AD, but present in all life stages. It was detected in the nucleoli of the enlarged muscle cell of the host within the capsule of the parasite, suggesting that it might be involved in the regulation of host muscle cells during capsule formation. The coagulation assay indicated that *T. spiralis* serine protease (TsSP) could inhibit blood coagulation and protect the NBL through the circulatory system of the host during infection (Gao et al., 2018). TsSP was detected in cuticle and stichocytes and recombinant protein was used for ELISA to evaluate its potential in serodiagnosis (Sun et al., 2018a). rTsSP-ELISA was more sensitive than ES-ELISA for detecting anti-*Trichinella* IgG in human patients and showed better specificity when tested against sera from individuals with other parasitic infections and healthy controls. In mice, rTsSP-ELISA detected specific anti-*Trichinella* IgG by 10 dpi. Another study used recombinant TsSP for ELISA to detect IgG in experimentally infected mice and pigs (Sun et al., 2019a). Anti-*Trichinella* antibodies were detected in all experimentally infected mice and cross-reactions were not found. There was no significant difference in antibody detection rate among mice infected with encapsulated *Trichinella* species. Also, specific IgG was detected in all infected pigs with no cross reactivity.

The potential of TsSP as a vaccine target against *Trichinella* infection was then investigated (Sun et al., 2018b). The mice immunized with rTsSP experienced 53 % reduction of AD and a 52 % reduction of ML burden in comparison with a control group. The vaccination of mice with rTsSP induced Th2 predominant immune response. Anti-rTsSP antibodies also inhibited the larval invasion of enterocytes and killed NBL and ML and decreased larval infectivity and development in the host by an ADCC mode. Another study examined the potential of TsSP as a vaccine *via* intranasal administration to mice, (Sun et al., 2019b). Intranasal vaccination with rTsSP elicited intestinal local sIgA response and a TsSP-specific systemic antibody response. Animals exhibited a 71 % AD reduction and a 62 % ML reduction following challenge. Vaccination with rTsSP reduced intestinal *T. spiralis* development and decreased female fecundity.

TsSP was used in the form of a recombinant protein and DNA vaccine (Xu et al., 2020a). Mice immunized with rTsSP elicited 48 % reduction in ML. The combined immunity of DNA prime and protein boost induced better immune responses and provided 69 % reductions in ML, indicating that mixed immunity could improve the muscle larvae reduction rate. The most recent publication used pigs as a vaccine animal model (Xu et al., 2021a). Pigs immunized with recombinant *T. spiralis* adult serine protease (rTs-Adsp) showed a 51 % reduction in ML burden compared to control groups. Also, rTs-Adsp induced a Th1-dominant mixed immune response characterized by elevated IgG, IgG subtypes, and cytokines (IFN-γ, IL-2, IL-4, and IL-10), along with increased proportions of CD4+ T cells, B cells, and neutrophils. As there are very few publications concerning anti-*Trichinella* vaccination using pigs, which is the most important *Trichinella* host, this research is of great importance. In another study mice were orally vaccinated with Ts-ADpsp DNA delivered by attenuated L. *plantarum.* Cytokine analysis showed increased IFN-γ, IL-4, and IL-10 levels in vaccinated mice, indicating mixed Th1/Th2 immune response. AD burden was reduced by 62 % in SP-IL-4 group and ML burden was reduced by 61 % in the same group (Xue et al., 2022).

This serine protease was also investigated for its therapeutic potential in inflammatory bowel disease (IBD) in a mouse model (Pang et al., 2020). rTs-ADSp-7 significantly alleviated colitis symptoms, including weight loss, inflammation, and damage to the intestinal lining. The therapeutic effects were likely attributed to its ability to shift the immune balance from a pro-inflammatory Th1/Th17 response to a regulatory Th2/Treg response. In the spleen of mice decreased levels of Th1- and Th17-related inflammatory cytokines (IFN-γ, TNF-α, IL-17) were detected, also increased levels of Th2- and Treg-related cytokines (IL-4, IL-10, TGF-β) and enhanced populations of Tregs and Th2 cells.

One more serine protease, investigated in several studies, was first identified in NBL and used for immunization and immunomodulation analysis. NBL-SP was used in the form of a DNA vaccine (Xu et al., 2017a). Immunized mice elicited partial protective immunity against challenge infections with *T. spiralis* as shown by 77 % reduction in ML. NBL-SP was also tested as a novel immunotherapy for IBD (Qu et al., 2020). Earlier NBL-SP inoculation significantly alleviated TNBS-induced colitis severity in mice by modulating inflammatory cytokine production, leading to reduced intestinal inflammation. NBL-SP likely inhibits Th1 and Th17 immune responses while enhancing the Th2 and Treg immune responses.

The immunomodulatory effects of a recombinant NBL-SP on DSS-induced colitis in mice was also determined (Long et al., 2021). NBL-SP reduced colitis severity when administered prior to disease induction. It alleviated colitis symptoms such as weight loss and histopathological damage to the colon. It reduced markers of intestinal inflammation, including shorter colon length and increased disease index. It also decreased the production of pro-inflammatory cytokines, increased anti-inflammatory cytokines in the colon and significantly reduced macrophage recruitment to the site of inflammation and suppressed macrophage activity.

*Trichinella* elastase is another serine protease. It was discovered to have an important role in the invasion of the host's enteral epithelium and promoting the larva intrusion of IECs, as strong and specific binding between *T. spiralis* elastase (TsEla) and IECs was identified. Recombinant protein was used for ELISA to evaluate its potential in serodiagnosis (Hu et al., 2020). In infected mice rTsEla-ELISA and ES antigen-ELISA showed 100 % detection rates for *T. spiralis*-specific IgG at 35 dpi. IgM and IgE were also detectable. In human patients, sensitivity of rTsEla-ELISA was slightly higher than ES-ELISA with similar specificity. Some minor cross-reactivity was detected in the case of paragonimiasis, clonorchiasis and sparganum-infected mice. The aim of another study was to assess the protective immunity induced by vaccination with Ela in a mouse model (Zhang et al., 2020a). The immunized mice exhibited a 52 % reduction in enteral AW and a 64 % reduction in ML after challenge infection. It was shown that the immune response protected enteral mucosa from larval intrusion, suppressed larval development and reduced female fecundity. Mice were orally vaccinated with TsE DNA delivered by attenuated *Salmonella* (Zhang et al., 2022). Vaccination elicited a systemic Th1/Th2/Treg mixed immune response and local enteral mucosal IgA response, but immune protection against *T. spiralis* larval challenge was similar to that induced by parenteral immunization - 52 % reduction of AD and a 69 % reduction of ML.

Some other serine proteases were described only once in the literature. *T. spiralis* TspSP-1.3 was cloned and expressed and western blot analysis indicated strong antigenic properties. Mice immunized with rTspSP-1.3 exhibited a humoral response and showed a 39 % reduction in ML burden compared to controls when challenged (Li et al., 2013). In the study by Yue et al. (2020) details of serine protease host interactions were also provided. Similarly to others, TsSerp aids larval penetration into IECs and the intestinal mucosa. Pre-incubating larvae with anti-TsSerp antibodies significantly reduced their ability to invade IECs. Anti-TsSerp antibodies likely form immune complexes on the larval surface, particularly at the anterior region, preventing larvae from directly contacting and penetrating host cells. Vaccination with recombinant TsSerp elicited strong systemic and mucosal immunity in mice, with high levels of IgG, IgG1, IgG2a, IgE, and IgM, indicating a mixed Th1/Th2 immune response. Vaccinated mice exhibited 49 % reduction in AD and 53 % reduction in ML burdens.

In another study mice were orally vaccinated with calreticulin and serine protease 1.1 delivered by attenuated *Salmonella* (Bai et al., 2022). Vaccination elicited a local gut mucosal IgA response and systemic Th1/Th2 mixed response. Mice immunized with TsSP1.1 alone exhibited 42 % reduction in intestinal AD and 29 % reduction of ML. Immune protection against *T. spiralis* larval challenge was higher when both proteins were used in immunization - 53 % reduction of AD and a 46 % reduction of ML. In the similar study, oral vaccination of mice with *Salmonella*-delivered rTsSP1.2 DNA vaccine, induced an intestinal mucosal IgA response and a systemic Th1/Th2 immune response (Li et al., 2018). The vaccinated mice showed a 33 % reduction of intestinal AD and 72 % reduction of ML after challenge.

A peptidase from *T. spiralis* was investigated for biological properties and functions, and protective immunity induced by immunization (Lei et al., 2020). rTsP triggered high levels of IgG, IgG1/IgG2a, and IgA, and elicited systemic and local intestinal mucosal cellular immune responses, as demonstrated by the cytokines IFN-γ and IL-4. Immunization of mice with rTsP reduced the numbers of intestinal AD by 39 % and ML by 42 %. *Trichinella* trypsin was investigated in a similar manner (Zhang et al., 2020b). Vaccination of mice triggered a humoral immune response and also systemic and local enteral cellular immune responses. The mice vaccinated with rTsT exhibited a 33 % reduction of enteral AD and a 38 % reduction of ML.

Another study aimed to evaluate a recombinant trypsin as a potential early diagnostic antigen (Han et al., 2024). ELISA using rTsTryp and ES antigens was performed to detect specific IgG and IgM antibodies in serum samples from infected mice, swine, and humans. It was found that Tryp-ELISA of mice sera detected specific IgG two days earlier compared to ES-ELISA. In human serum samples, Tryp-ELISA had a sensitivity of 98 % and specificity of 99 %, which was significantly higher than ML ES antigen-ELISA. Also, Tryp had lower cross-reactivity with other parasitic infections like clonorchiasis, cysticercosis, echinococcosis and *Ascaris suum*. Serum samples positive by Tryp-ELISA were also confirmed by western blot, which showed a single clear band.

The last study with serine protease reviewed here, aimed to evaluate a diagnostic assay specific to *Trichinella papuae*, a non-encapsulated clade of *Trichinella* species. The tissue-type plasminogen activator (PLAT) gene was cloned, expressed and purified. The recombinant protein was immunogenic and identified as a secretory enzyme. An indirect IgG-ELISA was developed using rTpaPLAT as the antigen. Testing showed 100 % sensitivity and 86 % specificity in detecting human *T. papuae* infections, no cross-reactivity with *T. spiralis* sera, but false positives occurred with other parasitic infections, such as strongyloidiasis, echinococcosis, and amoebiasis. Nevertheless, this was the first immunodiagnostic method with no cross-reactivity to *T. spiralis* (Sahaisook et al., 2022).

A number of serine proteases have been investigated as potential targets for vaccines against *T. spiralis*. Immunization of mice with recombinant single serine proteases only elicited a partial protection against challenge. The best results for reducing work burdens were obtained when a DNA vaccine was used in different configurations (61–77 %). Like other enzymes, protease activity of SP can be weakened *via* protein mutation to reduce its regulatory activity and increase vaccine efficacy. Interestingly, promising results (62 % protection) were also obtained when SP was delivered intranasally, emphasizing a key role of mucosal response against the intestinal trichinellosis phase. Therapeutic potential of SP for colitis treatment was initially evaluated and may be developed for Crohn's disease or other Th1 immunity-mediated diseases.

### 3.2 Cathepsins

Cysteine proteases are a superfamily of proteolytic enzymes in parasitic organisms involved in worm intrusion, migration and development, digestion and degradation of host proteins, and immune evasion. The cysteine proteases include cathepsin B, C, F, H, K, L, O, S, V, W and X. For example, cathepsin B is a member of the papain-like cysteine protease family and might be secreted into the extracellular matrix and bound to the cellular membranes. Some of them have been revealed to be potential drug targets for antihelminth agents, chemotherapy or immunoprophylaxis, vaccine antigen candidates as well as for use in immunodiagnostics (Grote et al., 2018).

*T. spiralis* cathepsin B (TsCB) was transcribed and expressed in three main *T. spiralis* life-cycle stages (AD, NBL, ML) and it was present on the parasite's surface, especially in the cuticle and stichosome. It might participate in intestinal intrusion of larvae in the enteral epithelium during *Trichinella* infection (Cui et al., 2019). Vaccination of mice with recombinant TsCB induced production of specific antibodies (IgG and IgE), demonstrating a Th2-skewed immune response. There was 53 % reduction in intestinal AD, 51 % reduction in ML burden and decreased worm fecundity and growth. Another immunization study of cathepsin B was performed (Yang et al., 2018). Vaccination of mice with TsCPB2 significantly increased total serum IgG levels compared to controls and induced a mixed Th1/Th2 immune response, significantly increasing both IgG and IgG2a levels and a mixed Th1/Th2 cytokine profile. Vaccination also markedly enhanced IgE levels, indicating its role in promoting intestinal worm expulsion. Mice vaccinated with TsCPB2 exhibited 52 % reduction in AD and 51 % reduction in ML burden.

Cathepsin B was also used in the study of immunomodulatory effects on intestinal ischemia/reperfusion (I/R) injury in a mouse model (Liu et al., 2015c). Mice were vaccinated subcutaneously with rTsCPB prior to I/R injury and then survival rates, histological analysis and immunological analysis were performed. Mice treated with rTsCPB exhibited higher survival rates post-I/R injury, reduced neutrophil infiltration in intestinal tissues, increased cell proliferation and restoration of body weight. I/R injury induced a shift from anti-inflammatory M2 to pro-inflammatory M1 macrophages and rTsCPB reversed this effect, promoting M1-to-M2 polarization through STAT6 signaling.

A recombinant cysteine protease belonging to the cathepsin B group was evaluated for immunogenicity, immune response, and protective efficacy against *T. spiralis* (Song et al., 2018a). Western blot and ELISA confirmed the antigenicity of rTsCP, and it was recognized by sera from infected mice. Mice vaccinated with rTsCP developed strong Th2-predominant immune responses and vaccination reduced AD by 54 % and ML burden by 33 % following challenge infection. Anti-rTsCP serum partially inhibited *in vitro* invasion of IECs by larvae and promoted the adherence of peritoneal macrophages to NBL, increasing larval death.

An interesting example of an immunization study was conducted using cathepsin X and aminopeptidase P. Cathepsin X is expressed in different *T. spiralis* stages, has the capacity to specifically bind with IECs, and promote larval invasion (Zeng et al., 2021). Silencing TsCX by specific siRNA impaired larval invasion, making it a potential vaccine target. Vaccination with rTsCX elicited elevated levels of specific serum IgG and enhanced gut mucosal sIgA response. It also induced a mixed Th1/Th2 immune response, indicated by increased IFN-γ and IL-4 cytokine levels. Mice immunized with TsCX alone exhibited 49 % reduction of intestinal AW and 55 % reduction of ML. Immune protection against *T. spiralis* larval challenge was higher when induced by immunization with cathepsin X and aminopeptidase – resulting in a 64 % reduction of AD and a 69 % reduction of ML. TsCX vaccination enhanced ADCC, leading to an NBL reduction of 33 %.

In another study, mice were orally vaccinated with cathepsin F DNA delivered by attenuated *L.* *plantarum* (Xue et al., 2021b)*.* The parasite's cathepsin F hydrolyses host proteins and plays an important role in the invasion of host tissues. Thus, it can be used as a potential vaccine target. Constructed vaccines effectively induced robust humoral and mucosal immune responses, including high levels of IgG and sIgA, as well as a mixed Th1/Th2 cytokine response. The invasive TsCPF1-IL-4 vaccine group provided the highest level of protective immunity against *T. spiralis* infection, reducing both AD and ML burdens significantly (47 % and 59 %), as well as lessening histopathological damage.

The *in vitro* modulatory effect of two cathepsins (L and C) was investigated. Cathepsin L is involved in disrupting the intestinal epithelial barrier and facilitates *T*. *spiralis* larval invasion. Cathepsin C is a secretory protein that is highly expressed in the infective larvae and AD stages in the intestines; it promotes larval invasion of IECs. In the first study cathepsin L was co-incubated with RAW264.7 murine macrophage cells. The results indicated that rTsCatL2 induces macrophage M1 polarization *via* the NF-κB pathway and enhances the ADCC killing of NBL as measured by ELISA, qPCR, Western blot, immunofluorescence and flow cytometry (Liu et al., 2023).

In the second study dipeptidyl peptidase 1 (cathepsin C) was co-incubated with RAW264.7 macrophages and murine peritoneal macrophages. Polarization was analyzed using similar methods and the results indicated that rTsDPP1 induced macrophage M2 polarization, upregulated the expression of anti-inflammatory cytokines, and inhibited macrophage-mediated ADCC *via* activation of the STAT6/PPARγ pathway (Yan et al., 2023). These studies provide novel insights into the mechanisms of immunomodulation and immune evasion by *Trichinella* during infection.

Cysteine proteases which include cathepsins B, C, F, L, and X didn't induce high levels of protection against *Trichinella*, even when delivered as DNA by a bacteria vector. The best result was in the case of cathepsin X combination with aminopeptidase (69 %), proving again that joining different antigens is a promising strategy. Similar to previous enzymes, protease activity of cathepsins can be weakened *via* protein mutation to reduce their regulatory activity and to increase vaccine efficacy. The therapeutic potential of cathepsins was barely explored and was only investigated *in vitro*, so to draw any conclusion, animal experiments have to be conducted.

### 3.3 Other proteases

Other *Trichinella* proteases that have been cloned and used as recombinant proteins are aminopeptidases and aspartyl proteases. Aminopeptidases have roles in survival, invasion, and nutrient acquisition. They may be involved in immune evasion and with the larval penetration of IECs and mediate or facilitate the entry into muscle cells. *T. spiralis* aminopeptidase (TsAP) was characterized and evaluated as a vaccine candidate (Zhang et al., 2013). Vaccinated mice developed specific antibodies, as evidenced by ELISA and Western blot analyses. Mice immunized with recombinant TsAP showed a 38 % reduction in AD burden and a 59 % reduction in ML burden following a challenge with *T. spiralis* larvae.

The immunization study using aminopeptidase P (TsAPP) and cathepsin X (TsCX) elicited elevated levels of specific serum IgG, an enhanced gut mucosal sIgA response and induced a mixed Th1/Th2 immune response (Zeng et al., 2021). Mice immunized with TsAPP alone exhibited 45 % reduction of intestinal AW and 50 % reduction of ML. Immune protection was higher when induced by immunization with both proteins – resulting in 64 % reduction of AD and a 69 % reduction of ML.

An aspartic protease-2 (TsASP2) which also plays a role in *T. spiralis* invasion of IECs, was used for an immunization study (Xu et al., 2021c). Vaccination with rTsASP2 induced significant immune responses, with a dominance of Th2 responses as indicated by elevated IL-4 levels. Mice showed increased serum IgG (especially IgG1) and intestinal secretory IgA levels post-vaccination. Vaccination resulted in 54 % reduction in intestinal AD at and 55 % reduction in ML burden.

Different aspartyl proteases was also used for an immunization study (TsASP1). Mice immunized with rTsASP1 elicited a robust humoral and cellular immune response. Elevated levels of rTsASP1-specific IgG, IgG1, and IgG2a were observed, as well as intestinal secretory IgA. Cytokine analysis revealed increased production of IFN-γ and IL-4 in spleen and mesenteric lymph node cells, demonstrating a mixed response with Th2 predominance. Immunization reduced AD and ML burdens by 52 % and 61 % respectively, upon challenge infection (Xu et al., 2021d).

The above mentioned proteases induce moderate levels of protection against *Trichinella* when delivered by conventional route (50–61 %). Combination of aminopeptidase with cathepsin X provided the best effect. As in the case of other proteases, enzyme activity may have an impact on protective efficacy.

## 4 Protease inhibitors

### 4.1 Cystatins

Cystatins are inhibitors of cysteine proteases and they play a role in regulating the proteolytic activity of the host and protect parasites against the immune system. They play an important role in helping the parasites evade host immune responses and adapt to a parasitic lifestyle. Protease inhibitors that have been studied are listed in Table 3. They are mostly small proteins, sharing a conserved structure essential for their inhibitory function. However, type III cystatins are larger multidomain proteins, usually containing three cystatin domains. Multi cystatin-like domain protein (CLP) from *T. britovi* was examined as a vaccine candidate against trichinellosis in mice (Stachyra et al., 2019). Recombinant antigen from *Trichinella* was produced in a eukaryotic expression system (*P. pastoris* yeast), which ensured post-translational modifications such as glycosylation and formation of disulfide bonds, what may be important for the activity of this protein. CLP induced a mixed Th1/Th2 immune response, measured by serum IgG, IgG1 and IgG2a antibodies and spleen IL-2, IL-4, IL-10, IFN-g levels. Partial protection against infection with *Trichinella* was observed after immunization and challenge in mice, the ML reduction rate was 47 %. Also, recombinant protein was used for ELISA to evaluate its potential in serodiagnosis in experimentally infected pigs and seroconversion was detected at 24 dpi.

**Table 3:** Recombinant *Trichinella* protease inhibitors.

Protein group	Name in the reference	Unified name	Accession number	Applicational context	ML reduction*	Reference
Cystatins	TbCLP	Cystatin	QTG10996.1^a^	Diagnostics, Vaccines, Immune modulation	46 %	(Stachyra et al., 2019; Stachyra and Wesolowska, 2023)
	Ts-CLP TS-cystatin	Cystatin	ABY60755.1^a^	Diagnostics, Vaccines	0–61 %	(Tang et al., 2015; Liu et al., 2021a; Liu et al., 2021b; Liu et al., 2014)
	TsCstN	Cystatin	XP_003369399.1	Immune modulation	na	(Kobpornchai et al., 2020; Kobpornchai et al., 2021; Thammasonthijarern et al., 2023)
	Ts-Cys	Cystatin	BQ692489.1	Immune modulation	na	(Li et al., 2022)
	Ts Cystatin	Cystatin	XP_003379766.1	Immune modulation	na	(Xu et al., 2019a)
Serpins	Tp-Serpin	Serpin	AEO72145.1	Immune modulation	na	(Xu et al., 2017c)
	TsSPI	Serine protease inhibitor	XP_003377380.1	Vaccines, Immune modulation	57 %	(Song et al., 2018a; Cortez-de-la-Fuente et al., 2023; Long et al., 2024)
	Ts-SPI TsKaSPI	Serine protease inhibitor	XP_003379899.1	Vaccines, Immune modulation	na	(Tong et al., 2024; Xu et al., 2018; Xu et al., 2020b; Xu et al., 2019b)1
	Ts-serpin TsAdSPI Serpin-type SPI TspAd5	Serpin	ABI32311.1^bc^ ABY60739.1^b^	Vaccines, Immune modulation	40–54 %	(Xu et al., 2017b; Jin et al., 2020b; Xu et al., 2018; Xu et al., 2020b; Xu et al., 2019b; Xu et al., 2021e)
	TsSERP1	Serine protease inhibitor	AAF63473.1^c^	Diagnostics, Immune modulation	na	(Lobanov et al., 2022; Kobpornchai et al., 2022)

* - reduction after single protein immunization, combination of two proteins is not shown;; ^a^ – sequences shares 93.04 % identity; ^b^ - sequences are identical; ^c^ – sequences shares 99.46 % identity.

A similar cystatin was used in studies on *Trichinella* serodiagnostics. In the first study CLP was used in a competitive ELISA (Liu et al., 2021b). The rCLP-cELISA demonstrated high sensitivity (96 %) and specificity (100 %) in human serum samples, accurate detection of infections in swine and mice samples, even at low infection levels, no cross-reactions with other parasites or viruses and versatility in diagnosing different *Trichinella* species. A second study evaluated the potential of CLP in serodiagnosis and identified its immunodominant B-cell epitopes using monoclonal antibodies to improve early diagnosis of *T. spiralis* infection (Liu et al., 2021a). Sera from experimentally infected swine and negative controls were used to validate the rTs-CLP ELISA. Monoclonal antibodies against rTs-CLP were generated, and immunodominant epitopes were identified using synthetic peptides and Western blotting. The rTs-CLP ELISA detected specific IgG antibodies as early as 17 dpi and showed sustained antibody detection up to 120 dpi. Also, two immunodominant epitopes were identified.

Another study on CLP serodiagnosis and immunization was performed (Tang et al., 2015). rTs-CLP was recognized by sera from hosts infected with *T. spiralis* and identified with a ∼ 46-kDa protein in ES products of various life stages. ELISA using rTs-CLP detected seroconversion in infected pigs as early as 15 dpi. Mice immunized with rTs-CLP showed 64 % reductions of AD and 61 % reduction of ML burden after challenge infection compared to controls. Additionally, female fecundity showed a 16 % reduction.

Mice were orally vaccinated with CLP delivered by attenuated *Salmonella* (Liu et al., 2014) inducing a mixed Th1/Th2 response, visible in Th1- and Th2-specific cellular transcription factors and the cytokine profiles, altered T cells and macrophage populations, and suppressed STAT6. Interestingly, AD recovery increased by 24 % in the immunized group, female fecundity decreased by 91 % and ML counts remained unchanged.

The immunomodulatory potential of recombinant CLP was analyzed *in vitro* by comparing changes of cytokines profiles of LPS-induced splenocytes (Stachyra and Wesolowska, 2023). A decrease in pro-inflammatory cytokines TNFα and IL-6 was observed after the administration of CLP compared to the LPS-stimulated group in total splenocyte populations, CD11b + splenocytes (monocytes), and CD11b- splenocytes (remaining cells). In contrast, the level of the regulatory cytokine IL-10 increased after CLP co-stimulation in CD11b- splenocytes and total splenocytes.

Several immunomodulation studies have been performed using type I small cystatins; one investigated the effect of recombinant cystatin on mouse bone marrow-derived macrophages (BMDMs). The novel recombinant *T. spiralis* cystatin (rTsCstN) internalized into mBMDMs within 60 min, primarily accumulating in the cytoplasm, although some co-localized with lysosomes (Kobpornchai et al., 2020). In LPS-stimulated mBMDMs rTsCstN significantly downregulated pro-inflammatory cytokines (IL-1β, IFN-γ, TNF-α), but it did not affect anti-inflammatory cytokine levels. Modulation of macrophage phenotypes by downregulating the M1 marker (iNOS) without affecting the M2 marker (Arg-1) was also observed. rTsCstN significantly suppressed MHC-II but did not significantly affect costimulatory molecules CD80 and CD86, suggesting selective immunomodulatory effects. A continuation study focused on how TsCstN affects the host immune response, in particular how it modulates macrophage and T cell interactions and suppresses IFN-γ production (Kobpornchai et al., 2021). *In vitro* experiments with BMDMs and T cells were used to study cytokine production and STAT4 signaling pathways. TsCstN suppressed the production of IFN-γ without affecting IL-17 A, selectively impairing Th1 responses while sparing Th17 pathways. It also reduced IL-12 production by macrophages, inhibited the phosphorylation and nuclear translocation of STAT4 and suppressed pro-inflammatory signals (nitric oxide and IL-6) in myotubules. The presented work identified potential pathways for ML to evade protective Th1-based immune responses and establish muscle-stage *T. spiralis* infection.

In the continued study, the therapeutic potential of recombinant cystatin was evaluated *in vivo* using an OVA-induced allergic asthma mouse model (Thammasonthijarern et al., 2023). Mice were parenterally sensitized and intranasally challenged with OVA to induce asthma. rTsCstN treatment was performed during sensitization and challenge. Positive effects on airway inflammation were observed, including reduction of eosinophil and macrophage infiltration in BALF and decreased immune cell infiltration in peribronchial and perivascular lung tissues. rTsCstN decreased levels of pro-inflammatory cytokines TNF-α and IFN-γ in BALF without altering regulatory cytokines. Treatment with rTsCstN also significantly lowered serum OVA-specific IgE levels, indicating reduced allergic sensitization.

Another experiment demonstrated that *T. spiralis*-derived cystatin had an effect on certain autoimmune diseases including IBD (Xu et al., 2019a). *In vivo* experiments were conducted on TNBS-induced colitis model in mice. TsCystatin was administered before TNBS treatment to evaluate its protective role and after TNBS-induced colitis to examine its treatment efficacy. It significantly reduced macroscopic and microscopic damage to the colon in mice. It also alleviated intestinal inflammation by decreasing pro-inflammatory cytokine levels and increasing anti-inflammatory cytokines and reduced the expression of NF-κB. TsCystatin shifted the immune response from a Th1-type to a Th2-type, increased the percentage of Tregs and immunosuppressive CD8 + CD28− T cells in the spleen. It decreased the CD4+/CD8+ T cell ratio, which might mitigate autoimmune responses. TsCystatin was more effective when administered before TNBS, indicating its stronger role in preempting IBD development.

The therapeutic effects of recombinant cystatin on polymicrobial sepsis in a mouse model was investigated (Li et al., 2022). Treatment with rTs-Cys significantly increased survival rates in septic mice, with 40 % surviving at 72 h, compared to 0 % in untreated septic mice. The treatment significantly reduced pathological injury in the lung and kidney and reduced numerous inflammatory markers. rTs-Cys suppressed the TLR2/MyD88 signaling pathway critical in sepsis-induced inflammation. Additionally, *in vitro* experiments have shown it shifts macrophage polarization from M1 to M2 phenotypes, characterized by reduced levels of pro-inflammatory cytokines and increased levels of anti-inflammatory cytokines.

Recombinant cystatins exhibit immunomodulatory properties and they are potential therapeutic agents for sepsis and other inflammatory diseases such IBD. However, attention should be paid to the effectiveness of therapeutic administration of proteins, not only prophylactic administration. In some studies, anti-inflammatory effects were observed when the proteins were administered prophylactically. Future research should focus on therapeutic effects. Cystatins are also proposed as a candidate for serodiagnosis. Cystatins didn't induce high levels of protection against *Trichinella* (46–61 %), and in the case of bacterial DNA immunization, CLP partially promoted infection, and no ML reduction was observed. It may be assumed that after intestinal delivery the protease inhibitor activity of CLP suppressed protective efficacy of the vaccine by regulating the host immune system. For using protease inhibitors, activity can also be weakened *via* protein mutation to reduce their regulatory activity and increase protectivity.

### 4.2 Serpins

Serpins, or serine protease inhibitors (SPI), are regulators of protease activity with broad implications in physiology, disease, and therapeutic development. Their primary function is inhibiting serine and cysteine proteases, but they also play roles in regulating inflammation by controlling the activity of proteases involved in inflammation, and immune modulation by influencing immune cell migration and cytokine release. The serpins released by helminths protect the worms from hydrolytic activity of host's serine proteases, help them to penetrate the defensive barriers, to escape immune attack, and favor the parasite's survival and colonization in the host (Dzik, 2006).

*T. spiralis* serpins are expressed in AD, NBL, ML of *T. spiralis*, with the ML stage demonstrating the highest expression level. These proteins not only play important roles in the process of *T. spiralis* molting and invasion of host cells, but they also regulate host anti-inflammatory immune responses (Yi et al., 2020). For this reason, most studies with serpins concern immunomodulation; however, others explore immunization and diagnostics applications. As an example, the immune protection induced by a SPI after immunization in mice was investigated (Song et al., 2018b). Vaccination with rTsSPI induced a strong humoral immune response and provided significant protection against *T. spiralis* infection. Specific IgG levels increased significantly after the second immunization. IgG1 levels were significantly higher than IgG2a, indicating a Th2-biased immune response. Immunization with rTsSPI resulted in 62 % reduction in intestinal AD burden and 57 % reduction in ML burden.

Investigation of the impact of different adjuvant formulations on the immune response in mice after recombinant serpin immunization was performed (Xu et al., 2017b). In immunized mice mixed immune responses were observed, with a Th2 predominance indicated by elevated IL-4 and IL-10 cytokines alongside IL-2 and IFN-γ. All three adjuvants used significantly enhanced the production of IgG, IgM, and IgE compared to the non-adjuvant treated group. The AD reduction rates in adjuvant treated groups were 62–52 %. Reduction in ML at 42 dpi was 46–36 %.

In a related study, other adjuvants were compared during mouse immunization with recombinant serpin (Jin et al., 2020b). NLRP3-deficient mice were used to assess the role of NLRP3 (a transcription factor in CD4+ T cells). Mice vaccinated with rTs-serpin exhibited high levels of IgG1, IgG2a, and IgE antibodies. A mixed Th1/Th2 immune response was induced, with elevated levels of IFN-γ and IL-4. The reduction rates were 54 % for AD and 55 % for ML. In NLRP3-deficient mice, the protective efficacy of rTs-serpin was significantly impaired.

The potential biomodulatory effects of recombinant serpin on macrophages derived from the THP-1 monocyte cell line were investigated using a recombinant protein produced in a *P. pastoris* yeast expression system (Cortez-de-la-Fuente et al., 2023).The THP-1 monocyte cell line was treated with serpin alone or in combination with M1/M2 stimuli. Recombinant serpin did not modulate IL-4, IL-10, IL-12p40, TNF-α cytokine levels or expression of CCR7, CD86 M1 and CD163, CD206 M2 markers. This suggests that this protein does not independently induce or interfere with macrophage polarization *in vitro*.

Another interesting study using serpin produced in *P. pastoris* evaluated the potential of an indirect ELISA with recombinant antigen as an alternative to the ES ELISA (Lobanov et al., 2022). The two listed publications are the only studies using a *Pichia* system besides the studies by Stachyra et al., 2019, Stachyra et al., 2020, and Grzelak et al. (2022) referred earlier. Diagnostic specificity and sensitivity were evaluated using sera from experimentally infected pigs and *Trichinella*-free commercial pigs. Both ES and serpin ELISAs achieved 98 % diagnostic specificity, but background reactivity was higher in the Serpin ELISA. Both assays demonstrated 100 % sensitivity, however the serpin ELISA showed delayed antibody detection compared to ES ELISA, particularly in pigs with low larval burdens. Also, the serpin ELISA demonstrated limited reactivity to other *Trichinella* species, suggesting potential as a screening tool for seroepidemiological studies of *T. spiralis* but being less effective for detecting other species.

An immunomodulatory study investigated the therapeutic potential of recombinant SPI in ameliorating colitis in a mouse model (Long et al., 2024), where mice were treated with SPI before colitis induction. The experiment demonstrated that SPI pre-treatment effectively ameliorated DSS-induced colitis by improving clinical symptoms, reducing inflammation and decreasing neutrophil infiltration into the colon, enhancing intestinal barrier integrity and promoting anti-inflammatory immune responses. Additionally, mice were immunized with SPI before experimental *Salmonella* infection to evaluate the protective effects of SPI on intestinal inflammation and bacterial translocation. Findings suggest that SPI could be a promising therapeutic agent, since it reduced clinical symptoms and tissue inflammation, lowered bacterial translocation and systemic spread, and preserved intestinal barrier integrity.

The therapeutic effects of a SPI on non-alcoholic fatty liver disease (NAFLD), a metabolic disorder often linked to obesity and inflammation, was investigated (Tong et al., 2024). In the study, evidence for the role of rTs-SPI in modulating inflammation, gut-liver crosstalk, and lipid metabolism to ameliorate NAFLD were provided using a mouse model of high-fat diet-induced NAFLD. SPI injections reduced liver steatosis, inflammation and fibrosis without affecting food intake. It also decreased macrophage infiltration in the liver, increased level of Tregs and suppressed TLR4/NF-κB/NLRP3 signaling. SPI enhanced intestinal barrier integrity, reducing endotoxin translocation to the liver, and improved gut microbiota composition by increasing beneficial bacteria. Additionally, SPI-treated mice were used as donors for fecal microbiota transplantation into antibiotic-treated recipient mice fed a high-fat diet (HFD). The experiment showed that fecal microbiota transplantation was effective in ameliorating NAFLD in recipient animals.

Another study explored the role of a serine protease inhibitor in modulating human neutrophil functions, particularly its ability to inhibit human neutrophil elastase (Kobpornchai et al., 2022). Human neutrophils were isolated from healthy donors and *in vitro* assays were conducted to assess *T. spiralis* secreted serine protease inhibitors (TsSERP1's) effects on neutrophil elastase activity, phagocytosis, neutrophil extracellular trap (NET) formation, and cytokine production. TsSERP1 preferentially inhibited neutrophil elastase, reduced phagocytic activity and suppressed NET formation. Additionally, TsSERP1 downregulated the pro-inflammatory cytokine (IL-1β, TNF-α, IL-6) and chemokine (IL-8, CCL3) secretion, diminishing the inflammatory response. rTsSERP1 suppressed the production of proinflammatory cytokines and chemokines during neutrophil activation, which are essential for neutrophil-mediated local or systemic inflammation regulation.

Two studies by Xu et al. (2018) investigated the immunomodulatory effects of recombinant *T. spiralis* serine protease inhibitors (TsKaSPI and TsAdSPI) on experimental colitis in mice induced by TNBS. The first focused on colonic pathological changes immune response and the second focused on macrophage activation in spleens and MLNs. In the first study SPIs significantly reduced colonic injury and inflammation in TNBS-induced colitis in mice and promoted a shift from a Th1-dominated immune response to a Th2-dominated one. An increase in Tregs was also observed, especially in the spleen and MLNs. The expression of NF-κB and the clinical symptoms were reduced in treated groups. In the second study, SPIs promoted the shift of macrophages from the M1 phenotype to the M2 phenotype. This shift was associated with a decrease in inflammation and improved tissue repair. The inhibitors also reduced pro-inflammatory cytokines (IL-23 and IL-12) and increased anti-inflammatory cytokines (IL-10 and TGF-β), enhanced activation of the IL-6/JAK2/STAT3 and IL-33/ST2 and reduced inflammatory markers. TsAdSPI consistently showed slightly stronger anti-inflammatory effects compared to the TsKaSPI (Xu et al., 2020b). A related study described immunomodulatory properties of TsKaSPI and TsAdSPI and their potential to protect hosts against *T. spiralis* infection (Xu et al., 2019b). Mice were immunized with TsKaSPI or TsAdSPI and challenged with *Trichinella*. Both SPIs induced higher levels of IgG1 and IgG2a in serum, indicating a mixed immune response dominated by Th2-type immunity, elevated levels of both pro-inflammatory cytokines and anti-inflammatory cytokines and increased Tregs and B regulatory cells in the spleen. Mice exhibited a significant reduction in AD, and the reduction rate was slightly higher for TsAdSPI than TsKaSPI and was 20–35 %. Additionally, stimulation of peritoneal macrophages with SPIs *in vitro* significantly increased secretion of cytokines compared to controls, but TsAdSPI induced slightly higher levels, compared to TsKaSPI. SPIs also induced significant phosphorylation of JAK2 and STAT3 signaling pathways, but TsAdSPI showed a slightly stronger activation.

The mechanism by which Ts-serpin mediates alternative activation of macrophages to create an anti-inflammatory environment was investigated (Xu et al., 2021e). Several experiments using mouse BMDMs indicated that serpin induced the polarization of macrophages into an alternatively activated phenotype, characterized by high expression of markers like CD206 and F4/80, up-regulation of anti-inflammatory cytokines IL-10 and TGF-β1 and downregulation of pro-inflammatory cytokines IL-1β and IL-12. This macrophage polarization was mediated *via* the STAT6 pathway, independent of IL-4 receptor alpha. In an *in vivo* experiment Ts-serpin or Ts-serpin-treated macrophages alleviated inflammation in a TNBS-induced IBD model in mice, demonstrating potential for therapeutic applications in inflammatory diseases. In the earlier study by Xu et al. (2017c), a serpin from *T. pseudospiralis* was investigated to reveal its role in macrophage polarization and immune evasion. The murine macrophage cell line J774A.1 was used in the experiment. rTp-serpin induced polarization of macrophages toward the M2 phenotype through activation of STAT3 signaling pathway, and inhibited LPS-induced M1 activation by inhibition of pro-inflammatory cytokines. M2 *in vitro* polarization was confirmed by flow cytometry and the up-regulation of M2-associated genes (cytokines and effector molecules). The results of this study are interesting since non-encapsulating species like *T. pseudospiralis* differ in some molecular mechanisms from encapsulating ones, such as inducing stronger immunosuppression.

TsSPIs may offer novel anti-inflammatory treatments for autoimmune diseases such as IBD, however several inconsistencies were observed during macrophage polarization *in vitro* experiments. In one study no effect on macrophages was observed, which is contrary to other studies. It would be advisable to repeat these experiments taking into account and comparing the differences in experimental procedures. As in the case of cystatins, more attention should be paid to the effectiveness of administration after induction of the disease. Serine protease inhibitors exhibited a partial protection against infection. Further testing of DNA vaccines, combination with other proteins and blocking their activity to reduce their immunomodulatory activity are needed.

## 5 Other proteins

### 5.1 Unknown *Trichinella*-specific proteins

A unique group of *Trichinella* proteins consists of proteins of unknown function. They have no conserved domains and no homology with other known proteins, but some are common in *Trichinella spp.* They are often identified as extracellular proteins with a signal peptide and are typically of low molecular weight. It is highly probable that they perform specific function in parasite-host interactions. Some of these unknown proteins were cloned and produced for investigation, although their roles remain unclear. They are listed in Table 4.

**Table 4:** Other recombinant *Trichinella* proteins.

Protein group	Name in the reference	Unified name	Accession number	Applicational context	ML reduction*	Reference
Unknown trichinella specific proteins	TbES21	21 kDa excretory/secretory protein	UPO81527.1	Diagnostics	na	(Grzelak et al., 2022)
	20 kDa Protein Ts-ES-1	21 kDa excretory/secretory protein	AAB48489.1	Vaccines	42 %	(Bi et al., 2015)
	SML-4	SML-4	QNN88866.1	Vaccines	47–56 %	(Srey et al., 2020)
	TPD52	Tumor protein D52	XP_003375379.1	Immune modulation	na	(Yue et al., 2021)
	SML-5	SML-5	QNN88867.1	Vaccines	0–43 %	(Srey et al., 2020)
	P53 Tsp53 TsP53	53-kDa protein	AAA97512.1^a^	Immune modulation		(Cvetkovic et al., 2016; Yang et al., 2016a; Chen et al., 2015; Chen et al., 2016; Wei et al., 2021)
	Tpp53	53-kDa protein	AAK29415.1	Immune modulation	na	(Khueangchiangkhwang et al., 2023)
	T653K	53-kDa protein	ABD66079.1^a^	Vaccines	53 %	(Lee et al., 2016)
Regularory, chaperons *etc.*	TsCRT	Calreticulin	KRY34215.1	Vaccines	35 %	(Bai et al., 2022)
	TsCRT Ts-CRT	Calreticulin	XP_003371379.1	Immune modulation	na	(Zhao et al., 2017; Shao et al., 2020)
	14–3-3	14–3-3 protein	QOP59256.1^b^ XP_003378934.1^b^	Vaccines	0–46 %	(Yang et al., 2016b; Stachyra et al., 2020)
	Tsgal	Galectin	XP_003381656.1^c^ EFV62290.1^c^	Vaccines, Immune modulation	52–58 %	(Zhang et al., 2023; Xu et al., 2022; Li et al., 2024)
	HSP20	Heat shock protein 20	UPO81528.1	Diagnostics	na	(Grzelak et al., 2022)
	HSP70	Heat shock protein 70	AAK85149.2	Vaccines, Immune modulation	22 %	(Zhang et al., 2018a)
	TsMIF	Macrophage migration inhibitory factor	AAL12629.1	Immune modulation	na	(Huang et al., 2022)
	TsPmy Ts-Pmy Pmy	Paramyosin	ABO09862.1	Vaccines, Immune modulation	34–55 %	(Yang et al., 2010; Wang et al., 2016; Wang et al., 2017; Gu et al., 2017; Hao et al., 2021; Zhang et al., 2024; Guo et al., 2016; Sun et al., 2015)
	Tspst	Proteasome subunit beta type-7	XP_003374439.1	Vaccines	na	(Yang et al., 2015)

* - reduction after single protein immunization, combination of two proteins is not shown; ^a^ – sequences shares 90.50 % identity; ^b^ - sequences are identical; ^c^ - sequences are identical;

An example of such protein is a small protein with a signal peptide named ES21 (21 kDa excretory/secretory protein), secreted by *Trichinella* stichocytes and present in the ES products of *T. spiralis* AD and ML. The study by Grzelak et al. (2022) identified recombinant *T. britovi* proteins as potential markers for improved serodiagnosis of *Trichinella* infections, particularly for species-specific detection of *T. britovi*. The protein was cloned and expressed in a *P. pastoris* yeast system, then ELISA assays were used to assess IgG antibody levels in sera collected from mice and pigs experimentally infected with *T. britovi* or *T. spiralis*. ES21 was highly specific to *T. britovi* (no response in *T. spiralis*-infected pigs) and detected infection at 36 dpi but in only half of the samples, showing maximum reactivity at 62 dpi. The results indicated that rTbES21 have diagnostic potential for trichinellosis in ELISA, but it is highly specific to *T. britovi* and may lack broad applicability.

The protective potential of ES21 has been shown in immunization studies of mice (Bi et al., 2015). High antibody levels were detected with predominantly IgG1 but also IgG2a, and increased IFN-γ, IL-2, IL-4, IL-5 cytokine secretion from stimulated splenocytes from immunized mice indicating a mixed Th1/Th2 immune response. AD burden was reduced by 27 % and ML burden was reduced by 42 %.

Several immunomodulation studies were performed using the 53-kDa glycoprotein (P53), a major component of *Trichinella* ES proteins of ML and AD and it was one of the first *Trichinella* proteins analyzed and cloned (Nagano et al., 2008). The effects of Tsp53 on DCs and T cell responses in *in vitro* stimulated rat bone marrow-derived DCs were investigated (Cvetkovic et al., 2016). Tsp53 induced a semi-mature DC phenotype, characterized by low MHC II expression, moderate CD86 expression and high ICAM-1 expression, indicating a tolerogenic DC phenotype. It induced a high IL-10/low IL-12p70 ratio, but did not induce TGF-β. Next, Tsp53-induced DCs, when co-cultured with naïve T cells, promoted T cell proliferation with immune response skewed toward Th2 (high IL-4, IL-10) and reduced IFN-γ, with minimal production of TGF-β. Also, weak MAPK activation was observed, suggesting Tsp53 contributes to immune modulation but may require additional factors for full efficacy.

Another *in vitro* study investigated whether the 53-kDa protein can activate M2 macrophages while suppressing M1 macrophage activation (Chen et al., 2015). TsP53 promoted M2 macrophage activation of BMDMs cultures in a dose-dependent manner and suppressed M1 macrophage activation. Additionally, levels of secreted pro-inflammatory TNF-α, IL-6, IFN-γ cytokines decreased while levels of anti-inflammatory IL-4, IL-13, IL-10, and TGF-β cytokines increased with TsP53.

Anti-inflammatory properties of TsP53 and M2 macrophage activation *in vivo* in a mouse model of endotoxemia were investigated (Chen et al., 2016). rTsP53 increased survival in endotoxemia mice and reduced systemic inflammation when TsP53 was received one hour after LPS injection. TNF-α, IL-6, IFN-γ were significantly reduced, while anti-inflammatory cytokines were significantly increased after rTsP53 treatment. Also, rTsP53 prevented liver and lung damage, necrosis, congestion, and leukocyte infiltration, and promoted M2 macrophage polarization, which was confirmed with flow cytometry, immunohistochemistry and gene expression analysis. The study shows that shift from M1 to M2 macrophages reduces organ damage and improves survival in sepsis and other inflammatory diseases.

Another study was conducted using a rat sepsis model and rat macrophages *in vitro* (Yang et al., 2016a). Recombinant 53-kDa protein significantly improved survival in sepsis-induced rats. The protective mechanism was linked to an increase in M2 macrophages, which help control excessive inflammation. The experimental group showed a higher proportion of M2 macrophages. Decreased pro-inflammatory IFN-γ and TNF-α cytokines levels in the experimental *vs.* control groups were observed as were increased anti-inflammatory IL-4 and IL-13 cytokines levels in the experimental *vs.* control groups, indicating an immune shift from a Th1-dominant response to a Th2-dominant response.

The protective effects of TsP53 on acute lung injury (ALI) and its role in immune modulation *via* macrophage polarization was investigated in another study (Wei et al., 2021). ALI was induced in mice by intravenous injection of LPS and TsP53 was administered before LPS. rTsP53 significantly reduced pathological scores and pulmonary edema in treated mice, increased IL-4, IL-10, and IL-13 anti-inflammatory cytokines and decreased TNF-α, IL-6, and IL-1β pro-inflammatory cytokines in BALF. Also, M2 macrophage polarization was promoted, while M1 macrophages were suppressed and lung pyroptosis markers were downregulated in treated mice.

The most recent study investigated the immunomodulatory effects of *T. pseudospiralis* Tpp53 on psoriasis-like skin inflammation (Khueangchiangkhwang et al., 2023). It was aimed to determine whether Tpp53 can suppress inflammation by modulating the IL-23/IL-17 axis in a psoriasis mouse model, where rTpp53 was applied topically. Mice treated with rTpp53 exhibited fewer disease symptoms and less inflammatory cell infiltration in the skin. Treatment significantly reduced the expression of IL-17 A, IL-6, and IL-23 in psoriatic skin lesions and pro-inflammatory chemokines, antimicrobial peptides and β-defensins. Tpp53 suppressed IL-17 A production in splenocytes and IL-6 production in macrophages, suggesting a systemic effect beyond the skin.

An interesting study used the 53-kDa *Trichinella* protein in the form of virus-like particles (VLP), a new vaccine technology (Lee et al., 2016). VLPs containing P53 were produced in baculovirus expression system, a eukaryotic system using insect cells which allows for proper protein folding. IgG, IgG1, and IgG2a responses were significantly increased in VLP-immunized mice, with IgG2a being dominant before infection. Both Th1 (IFN-γ, IL-2) and Th2 (IL-4, IL-10) cytokines were elevated in vaccinated groups. P53 VLPs reduced ML burden by 34–53 %.

Another study examined the immunogenicity and protective efficacy of two novel *T. spiralis* proteins, SML-4 and SML-5, in mice (Srey et al., 2020). They are novel molecular weight proteins (15 kDa and 12 kDa), with no similarity to any sequences. These proteins are secreted during the intestinal phase; SML-4 is expressed in both ML and AD, whereas SML-5 is found only in ML in early stages of intestinal invasion. SML-4 and SML-5 induced IgG responses and both proteins elicited mixed Th1/Th2 responses, as shown by the presence of IgG1 and IgG2a isotypes. Splenocytes from vaccinated mice were collected and stimulated with recombinant SML-4 or SML-5 indicating that SML-4 vaccination induced a Th1-skewed response (high IFN-γ) and SML-5 vaccination induced a mixed Th1/Th2 response (both IFN-γ and IL-4). The ML were reduced by 47–56 % in the case of SML-4 and by 43 % in the case of SML-5 for moderate-dose challenge, and by 47 % in the case of SML-4 and no significant reduction in the case of SML-5 for high-dose challenge. Vaccination also led to 67 % reduction in AD for SML-4 and 48 % reduction for SML-5. Although the biological functions of SML-4 and SML-5 remain unknown, each protein may be important for establishment of parasite infection of the intestine, making them prophylactic vaccine candidates.

In recent years several studies confirmed a negative correlation between certain parasitic infections and the incidence of tumors (Callejas et al., 2018). It was also confirmed that *T. spiralis,* in addition to its immunomodulatory properties, can induce antitumor immunity (Wang et al., 2013). The last, unique study investigated whether antibodies against TPD52 *Trichinella* protein, can inhibit osteosarcoma cell proliferation and tumor growth, as *Trichinella* has reported anti-tumor properties (Yue et al., 2021). It is known that some parasites share cross antigens with tumors, and those antigens may help break immune tolerance and induce immune responses against cancer. TPD52 is a cross antigen between *T. spiralis* and osteosarcoma. MG-63 osteosarcoma cells and osteosarcoma-induced mice were treated with TPD52 antiserum, obtained by immunization of mice with recombinant TPD52. The study revealed that TPD52 antiserum exhibits strong anti-osteosarcoma effects, primarily by inducing apoptosis and activating the immune response. *In vitro*, TPD52 antiserum inhibited osteosarcoma cell proliferation, while *in vivo* it significantly reduced osteosarcoma tumor growth in mice. Tumor volume was reduced by 62 % and tumor weight was reduced by 60 %.

Assessing potential applications for proteins of unknown functions is a great challenge, however promising findings are available. Moderate levels of protection were induced against *Trichinella* when delivered by conventional route (42–56 %) and VLPs (53 %). Combination of these proteins with other antigens and testing DNA vectors is advisable. P53 showed significant immunomodulatory properties, namely, to induce M2 polarization of macrophages, and alleviate inflammatory diseases like sepsis, psoriasis and ALI. TPD2 had unique antitumor properties and further *in vivo* studies exploring these features and mechanisms are needed.

### 5.2 Regulatory, chaperons and structural proteins

The final group of proteins discussed in this review included various regulatory and signaling proteins, chaperons and structural proteins, which are immunoreactive, present in ES products or in cuticle, or are of interest for other reasons. One of the most often investigated was paramyosin (Pmy), a structural protein found in the thick myofilaments of invertebrate muscle. It plays a crucial role in muscle contraction by providing structural stability and modulating the function of myosin. Pmy is also present on the parasite surface at different stages and was found in ES products of AD and ML. In an early study paramyosin was used for vaccine experiments (Yang et al., 2010). Mice were immunized with rTs-Pmy formulated with different adjuvants and immunization induced high levels of IgG antibodies. The IgG1/IgG2a ratio indicated a balanced Th1 and Th2 immune response, with a slight Th2 dominance. Spleen cells from immunized mice produced greater amounts of IFN-γ, IL-2, IL-4, and IL-10 compared to controls. Immunized mice showed 34–37 % ML reduction.

In another study mice were orally vaccinated with paramyosin delivered by attenuated *Salmonella* (Wang et al., 2016). Immunized mice exhibited high levels of IgG and IgA antibodies. IgG2a was the dominant subclass, indicating a Th1-biased response. Splenocytes and MLN cells from vaccinated mice showed elevated IFN-γ, IL-2, IL-4, IL-5, IL-6, IL-10 cytokines, indicating a mixed response. CCR9 and CCR10 receptors were highly expressed on B cells from vaccinated mice and 45 % reduction in AD burden and 47 % reduction in ML burden was detected after challenge.

Attempts to improve vaccine efficacy have used a heterologous prime-boost strategy, combining a TsPmy vaccine delivered *via* attenuated *Salmonella* with boosts of recombinant TsPmy protein (Wang et al., 2017). Heterologous prime-boost immunization resulted in higher IgG titers than DNA- or protein-only regimens and balanced Th1/Th2 responses. Mice in the DNA + protein and DNA-only groups produced high levels of specific sIgA, while the protein-only group did not. Heterologous prime-boost immunization induced the highest levels of both Th1 and Th2 cytokines, while DNA-only vaccination led to a stronger Th1 response, and protein-only vaccination induced a dominant Th2 response. The heterologous prime-boost group had the highest AD reduction (42 %) and ML reduction (55 %). The DNA-only group had 45 % AD reduction and 47 % ML reduction, while the protein-only group had 10 % AD reduction and 37 % ML reduction.

A novel approach was proposed in a study where a recombinant multi-epitope vaccine was created using four CD4+ T cell epitopes and one B cell epitope from Pmy (Gu et al., 2017). These epitopes were chosen based on prior research demonstrating their ability to induce protective immune responses. Immunized mice showed high IgG and IgG1/IgG2a ratios, suggesting a mixed Th1/Th2 response. Immunization induced significant T cell proliferation and elevated IFN-γ, IL-4, and IL-5 secretion. Also, splenocytes from vaccinated mice responded to synthetic peptides representing T cell epitopes, indicating efficient epitope presentation. Vaccination led to a 55 % reduction in ML, while a 34 % reduction was observed with whole Pmy immunization.

Paramyosin also serves as a potential modulator of the host immune system by interacting with human collagen, calgranulin *etc.* Pmy binds to human complement C1q, specifically to the A chain (Sun et al., 2015). *In vitro* experiments revealed that this binding inhibits classical complement activation, leading to reduced C3 deposition and impaired hemolysis of antibody-sensitized red blood cells. Also, Pmy prevents C1q from binding to IgM and interferes with C1q-mediated THP-1 macrophage binding and migration, suggesting a role in suppressing immune cell recruitment to infection sites. TsPmy's role in interacting with DCs and its impact on the immune system was explored *in vitro* (Guo et al., 2016). Pmy was produced in a baculovirus insect cell expression system, which allows for eukaryotic protein folding. Incubation with rTsPmy activated mouse bone marrow-derived DCs to a semi-mature state. They secreted cytokines associated with Th1, Th2, and Treg responses, including IL-1β, IL-6, IL-12p70, IFN-γ, TNF-α, and TGF-β. The DCs stimulated *T. spiralis*-sensitized CD4+ T cells, leading to increased proliferation and secretion of IL-4, IL-10, TGF-β, and IL-17 A, when co-cultured. They also promoted naïve CD4+ T cells to differentiate into Tregs.

It was also investigated whether TsPmy can enhance colonic Tregs and mitigate inflammation in mouse models of colitis (Hao et al., 2021). TsPmy significantly alleviated weight loss, diarrhea, and colonic inflammation in acute DSS-induced colitis when mice were treated intraperitoneally with recombinant TsPmy. Thymic-derived Tregs were promoted in the colonic lamina propria and IL-10 and TGF-β were increased while pro-inflammatory cytokines were reduced. Also, transfer of Tregs isolated from TsPmy-treated mice into Rag1 knockout mice with chronic colitis significantly reduced colonic inflammation.

A recent study investigated whether Ts-Pmy can modulate CD4+ T cell differentiation and alleviate RA in mice (Zhang et al., 2024). Ts-Pmy-treated mice had lower arthritis scores, less joint inflammation, and delayed disease onset compared to untreated mice, but prophylactic treatment (receiving Ts-Pmy injections before RA induction) was more effective than therapeutic administration (receiving Ts-Pmy injections after disease onset). Ts-Pmy significantly reduced Th1 and Th17 cell populations, increased Treg cells and lowered levels of pro-inflammatory cytokines IFN-γ and IL-17 A. An *in vitro* experiment with bone marrow-derived DCs indicated that Ts-Pmy inhibited Th1 and Th17 differentiation when presented by DCs and promoted Treg generation. This study suggests that *Ts*-Pmy may ameliorate RA by modulating CD4+ cell differentiation and provided new insights into the mechanism through which helminth-derived molecules exert their effects on autoimmune diseases.

Another protein that has been broadly studied is galectin (Gal). Gals are multifunctional proteins acting in immune regulation, cancer, apoptosis and pathogen interactions. They are involved in recognizing glycans on cell surfaces and extracellular matrix proteins In *Trichinella* they are expressed in all developmental stages, and primarily distributed on the surfaces, cuticles, stichosomes and embryos. Protein binds to IECs and can mediate invasion. Mice were orally vaccinated with Gal delivered by recombinant L. *plantarum* and some mice also received α-lactose to block Tsgal binding (Xu et al., 2022). High levels of IgG, IgG1, IgG2a, and IgA were detected in vaccinated mice. Significant increases of IL-4 and IFN-γ levels indicated a mixed Th1/Th2 response. No significant immune enhancement was observed with additional α-lactose. Gal vaccination significantly reduced parasite burden; 57–64 % reduction in AD and 53 %–59 % reduction in ML was observed. Interestingly, α-lactose alone also reduced worm burden ∼30 %, likely by interfering with Gal function.

A second study focused on evaluation of possible novel adjuvants for *Trichinella* Gal immunization (Zhang et al., 2023). Immunization induced specific IgG and IgA responses, increased Th1 (IFN-γ) and Th2 (IL-4) cytokine levels and reduced pro-inflammatory cytokines TNF-α, IL-1β. Protective efficacy against challenge revealed 40–51 % AD reduction and 38–52 % ML reduction.

Another recent study investigated whether recombinant *Trichinella* Gal can alleviate DSS-induced colitis in mice (Li et al., 2024) and explored its role in regulating gut microbiota. rTs-gal treatment reduced weight loss, diarrhea, and colon shortening in colitis mice as well as reduced colon inflammation, restored goblet cells, and improved mucus secretion. rTs-gal suppressed the TLR4/MyD88/NF-κB pathway, leading to lower levels of pro-inflammatory cytokines IL-6, IL-1β, and TNF-α. While DSS-induced colitis disrupted the gut microbiota, increasing harmful bacteria, rTs-gal treatment helped restore microbiota composition by increasing beneficial bacteria. This shift likely reduced LPS production, resulting in reduced gut inflammation.

The regulatory protein calreticulin (CRT) is a calcium-binding protein found in many parasites; it can interact with host immune components, including complement C1q. Mice were orally vaccinated with CRT and serine protease delivered by attenuated *Salmonella* (Bai et al., 2022)*.* Vaccination elicited a local gut mucosal sIgA response and systemic Th1/Th2 mixed response. Mice immunized with CRT alone exhibited 44 % reduction of intestinal AD and 35 % reduction of ML. Immune protection against *T. spiralis* larval challenge was higher when induced by both proteins - 53 % reduction of AD and a 46 % reduction of ML.

CRT interactions with human complement protein C1q was investigated, focusing on complement inhibition and macrophage modulation (Zhao et al., 2017). TsCRT bound to human C1q, specifically targeting its B chain and this interaction inhibited the classical complement activation pathway, what prevented complement-mediated parasite destruction. Also, TsCRT inhibited C1q binding to macrophages, reducing their ability to respond to infections, migration and the release of reactive oxygen intermediates. In another study full length the TsCRT and C1q-binding domains inhibited C1q-mediated neutrophil migration, reactive oxygen species release, and elastase secretion. TsCRT prevented NET formation, while NETosis triggered by C1q and immune complexes was significantly reduced. NET-related proteins, such as elastase and histone H3, were also reduced, indicating lower inflammatory responses. CRT may have potential therapeutic applications for complement-related autoimmune diseases (Shao et al., 2020).

Another study looked at 14–3–3, a regulatory adapter protein that plays important roles in numerous cellular processes and has been considered a potential vaccine candidate against parasitic infections. 14–3–3 regulates enzyme activity and the subcellular localization of target proteins, and plays roles in a variety of signaling pathways, including metabolism, cell division, stress responses, protein tracking and immune responses. To study the potential of the *T. spiralis* 14–3–3 protein as a vaccine candidate, mice were vaccinated with recombinant Ts14–3–3 protein (Yang et al., 2016b) High levels of total IgG and a balanced IgG1/IgG2a response were detected, indicating a mixed immune response. Splenocytes were isolated from vaccinated mice and stimulated *in vitro*, showing a significant increase in Th1 cytokines IFN-γ, IL-2 and Th2 cytokines IL-4, IL-5. The vaccinated mice showed a 46 % reduction in ML burden.

In a related study, mice were vaccinated with recombinant *T. britovi* 14–3–3 protein produced in a *P. pastoris* yeast expression system (Stachyra et al., 2020). Vaccination induced high levels of specific IgG and balanced IgG1/IgG2a levels, indicating a mixed Th1/Th2 immune response. Splenocytes were isolated from vaccinated mice and stimulated *in vitro*, showing a significant increase in Th1 cytokine IFN-γ and Th2 cytokines IL-4, IL-10. However, no significant reduction in ML burden was observed after challenge. Additionally, the recombinant protein was not well recognized by sera from infected mice, suggesting differences between native and recombinant protein epitopes.

Other studied proteins include heat shock proteins (HSPs), highly conserved and ubiquitously distributed proteins whose expression is induced by environmental changes and different forms of stress. These proteins have been classified into several families according to their molecular weights. HSP70 is the most conserved HSP present in all organisms and is an immunodominant antigen during *Trichinella* infections. It is also known to play key roles in immune activation. A study investigated whether TsHSP70 can activate DCs through TLR2 and TLR4, leading to protective immunity (Zhang et al., 2018a). Wild-type (WT), TLR2^−/−^, and TLR4^−/−^ mice were immunized with Ts-Hsp70 and challenged. WT mice had high IgG titers, while TLR2^−/−^ and TLR4^−/−^ mice had significantly lower IgG levels. Splenocyte proliferation and cytokine responses were also significantly reduced in TLR2^−/−^ and TLR4^−/−^ mice. Immunization reduced ML burden by 23 % in WT mice, while protection was significantly lower in TLR2^−/−^ (14 %) and TLR4^−/−^ (4 %) mice. An *in vitro* DC activation experiment confirmed that HSP70 activated dendritic cells *via* TLR2 and TLR4, and TLR4 plays a dominant role.

HSP20 was investigated in a study that identified recombinant *T. britovi* proteins as potential markers for improved serodiagnosis of *Trichinella* infections (Grzelak et al., 2022). Protein was cloned and expressed in *P. pastoris*, next ELISA assays were used to assess IgG antibody levels in sera collected from mice and pigs experimentally infected with *T. britovi* or *T. spiralis*. Recombinant HSP20 lacked stability, limiting its potential application.

Another protein that was used in immunization studies is proteasome subunit beta type-7 (Pst) (Yang et al., 2015). It is a structural and functional component of the proteasome that plays a supporting role in protein degradation. In parasites, the proteasome is involved in the regulation of cell differentiation and replication, and participation in impairment on hosts. Mice immunized with rTspst showed a 46 % reduction in AD burden after challenge infection with *T. spiralis*. Additionally, an *in vitro* invasion assay with IECs and ML demonstrated that Tspst is an invasion-related protein that plays a role in the early infection stages*.*

The last study with a regulatory protein reviewed here used a macrophage migration inhibitory factor (MIF) homolog from *Trichinella*, a protein that is a pleiotropic inflammatory mediator involved in host innate and adaptive immune responses to infection, acting as a cytokine, a chemokine, a hormone and an enzyme (Huang et al., 2022). Parasitic nematodes can use their MIF homologs to modulate the host immune response, favoring their invasion and maintenance. TsMIF was secreted by ML and bound to host monocytes *in vitro*. It bound CD74, the MIF host cell surface receptor, induced ERK1/2 phosphorylation, a key signaling event in immune cell activation. It promoted monocyte proliferation, similar to host MIF, but with weaker activation. It also interacted with a host intracellular protein Jab1, a coactivator of AP-1 transcription, which regulates cell proliferation and immune responses.

Regulatory proteins didn't induce high levels of protection when delivered by conventional routes (23–47 %). Slightly higher reduction rates (up to 59 %) were observed when novel approaches were used, as DNA prime/protein boost, multiepitope vaccine, *Lactobacillus* vector and combination of two proteins. The immunization strategy using these proteins requires careful consideration. Several proteins revealed significant immunomodulatory properties, especially to inhibit complement activation and may have potential to treat complement-related autoimmune diseases. Paramyosin has a dual role as a structural and immune-interacting protein and was shown to alleviate colitis and RA, while galectin was shown to also alleviate colitis. More *in vivo* studies are needed, as *in vitro* studies on monocytes, neutrophils, DCs, macrophages *etc.* are very promising.

## 6 Summary

Over the last decade, numerous diverse and innovative studies on *Trichinella*-derived recombinant proteins have been conducted. This review focuses on their potential applications, highlighting three main areas of research: diagnostics, vaccines, and therapeutics. The need for novel diagnostic assays for trichinellosis is significant, as current ELISA tests rely on ES antigens from *T. spiralis* ML, which have several limitations. These include complexity in preparation, low sensitivity in early infection detection, potential false positive results, and cross-reactivity with other parasite species. Recombinant proteins have been considered a promising alternative for developing a more accurate and early serological diagnostic test for both human and animal trichinellosis. Although several approaches have been reported, utilizing recombinant proteins such as ES21, cystatin, nudix hydrolase, serine proteases, and serpin in ELISA tests, no commercial test has been released to date. It is obvious that initial conclusions drawn from published studies were overly optimistic, while subsequent research revealed certain challenges. These included lower-than-expected sensitivity and specificity and a lack of significant advantages over traditional tests. It remains unclear what improvements could enhance the potential of recombinant antigen-based ELISA tests. One possible approach could be the combination of multiple proteins into a multi-antigen test; however, no such research has been published so far.

A similar situation exists with the development of a *Trichinella* vaccine. Despite extensive research, no effective formulation has been developed so far and identifying potential vaccine candidates for trichinellosis remains a significant challenge. This complexity arises from the fact that parasites are multicellular organisms with unique life cycles, inducing immune responses that differ from those triggered by other pathogens. Due to the intricate life cycle of *Trichinella*, the diversity of stage-specific antigens, and the parasite's immune-evasion strategies, its ability to evade vaccine-induced immunity is considerable. These factors make achieving effective protection against *Trichinella* invasion particularly difficult.

**Fig. 2:**
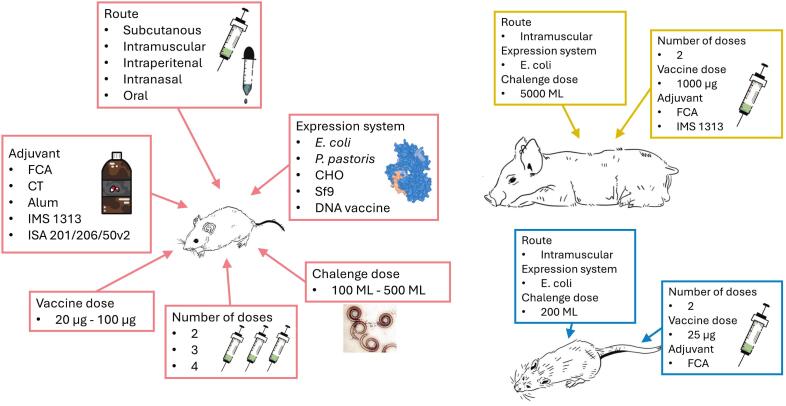
Summary of vaccine experiments using recombinant *Trichinella* proteins.

Due to these challenges, a “traditional” vaccine against Trichinella has never been developed. Instead, hopes have shifted toward next-generation vaccines utilizing recombinant proteins, DNA, viral vectors, and other advanced approaches. However, research reviewed in this study has yielded highly variable results in terms of protection against experimental infection. None of the experiments achieved complete or even 90 % protection and an average reduction was approximately 49 %. In two studies, no ML reduction was observed. Additionally, identifying the most promising vaccine candidate protein remains difficult due to significant differences in experimental methodologies. In the referenced mouse studies, protein doses ranged from 20 to 100 μg, with 20 μg being the most commonly used. The total number of doses varied between two and four, with a three-dose schedule being the most frequent. Furthermore, proteins were administered with or without adjuvants and through different routes (intramuscular, subcutaneous, intraperitoneal, intranasal). The experimental challenge involved 100–500 ML, with 300 ML being the most commonly used dose (Fig. 2). In DNA vaccination experiments doses of 50–100 μg DNA or 1 × 10^8^–1 × 10^9^ bacterial cells were used, administered intramuscularly or orally. These differences complicate direct comparisons of the protective potential of *Trichinella*-derived proteins - especially since none of them has demonstrated outstanding efficacy. To identify proteins with greatest potential, immunization experiments comparing proteins must be conducted using the same experimental conditions.

It is worth emphasizing that combining two proteins or using heterologous immunization with two different vaccine forms has shown promising results. Also, promising results were observed when DNA vaccine was delivered to the intestine *via* a bacterial vector and induced local mucosal response. Immunization experiments combining AP with cathepsin X, calreticulin with SP, two different DNases II, or DNase II with CTR resulted in higher protection against infection compared to single-protein immunization. Unfortunately, no studies have explored the use of three or more proteins in combination. Additionally, a heterologous prime-boost strategy using paramyosin, SP, and DNase II demonstrated significantly higher protection when DNA immunization was administered before the recombinant protein. These examples suggest that a multi-component vaccine approach would be more effective than a single-protein vaccine. As of today, no follow-up studies have been conducted in target hosts such as pigs, nor have any preclinical or clinical trials been reported. Only two small-scale pig studies have been published, achieving protection rates of 45 % and 50 %. These results remain insufficient for the commercial implementation of a vaccine. A multi-antigen vaccine strategy could be key to improving efficacy and making the vaccine viable for practical use. Another potential approach involves utilizing more advanced vaccine platforms, such as RNA-based vaccines, novel vectors and genetic engineering limiting enzymatic activity of proteins used. While these possibilities exist, the crucial question remains - will they be experimentally verified?

**Fig. 3:**
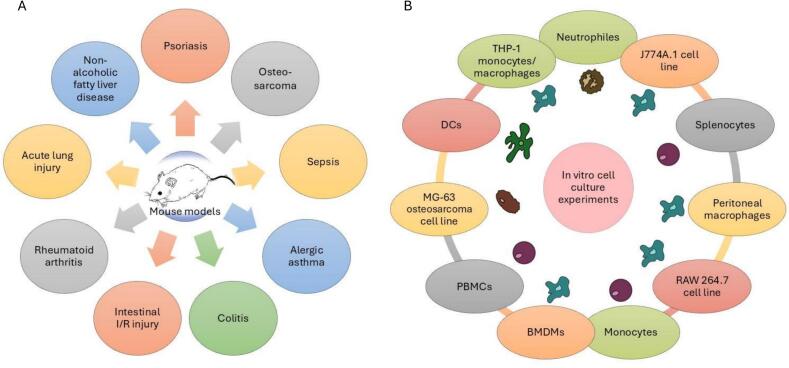
Summary of *in vivo* (A) and *in vitro* (B) immunomodulation experiments using recombinant *Trichinella* proteins.

The third area of research focuses on immunomodulation and the potential therapeutic applications of *Trichinella*-derived proteins, particularly for inflammatory and autoimmune diseases. As this field is relatively new and rapidly evolving, there is growing evidence that some of these proteins could have therapeutic value. *Trichinella* is a potent immunomodulator, and numerous studies have explored its potential *in vitro* and *in vivo*. However, a significant portion of this research has focused on ES products or on the effects of *Trichinella* infection itself. This review, however, includes only studies on recombinant proteins, omitting those involving ES products or live worms. Recombinant proteins such as cathepsin B, cystatins, TPD52, DNase II, galectin, P53, paramyosin, serine proteases, serpins, and SUCLA-β have demonstrated immune-modulating properties and have been shown to alleviate disease symptoms in mouse models of conditions that pose significant challenges to modern medicine (Fig. 3A). The experimental results have been highly promising. Additionally, several other recombinant proteins have been tested *in vitro* in immune cell culture experiments, including calreticulin, cathepsin L, dipeptidyl peptidase, HSP70, MIF and thioredoxin peroxidase (Fig. 3B). Further *in vivo* studies on these proteins are expected to be published in the future. Nevertheless, several challenges have already been identified. In some cases, the strongest anti-inflammatory effects were observed when the proteins were administered prophylactically. While this is valuable, it does not align with the primary therapeutic goal - treating existing immune disorders rather than preventing their onset. As demonstrated, numerous challenges remain in the application of recombinant proteins for therapeutic use, but continued research in this area holds great potential for future medical advancements.

While *Trichinella*-derived recombinant proteins hold significant promise in diagnostics, vaccine development, and therapeutics, substantial hurdles remain. For diagnostics, multi-antigen approaches may improve test performance. In vaccine research, heterologous immunization strategies, genetic engineering and advanced platforms could enhance protection. In therapeutics more studies are needed to transition from experimental models to clinical applications. Future research should focus on refining these approaches to unlock their full potential.

## CRediT authorship contribution statement

**Anna Stachyra:** Writing – review & editing, Writing – original draft, Visualization, Supervision, Software, Formal analysis, Data curation, Conceptualization. **Justyna Bień-Kalinowska:** Writing – review & editing, Writing – original draft, Supervision.

## Declaration of competing interest

The authors declare that they have no known competing financial interests or personal relationships that could have appeared to influence the work reported in this paper.

## Appendix A Supplementary data

Sequence aligments of cloned *Trichinella* recombinant proteins with common names. Aligments were made using Clustal Omega and visualized with ESPript 3.0 online software. Image 1
