# Supplementary material for: Assessing diagnostic, vaccine and therapeutic potential of selected Trichinella proteins
Source: Food Waterborne Parasitol. 2025 Sep 1;40:e00283. doi: 10.1016/j.fawpar.2025.e00283 (PMC12451359; doi:10.1016/j.fawpar.2025.e00283)

Supplementary file. Aligments of cloned *Trichinella* recombinant proteins with common names. Aligments were made using Clustal Omega and visualized with ESPript 3.0 online software.

A) DNases II

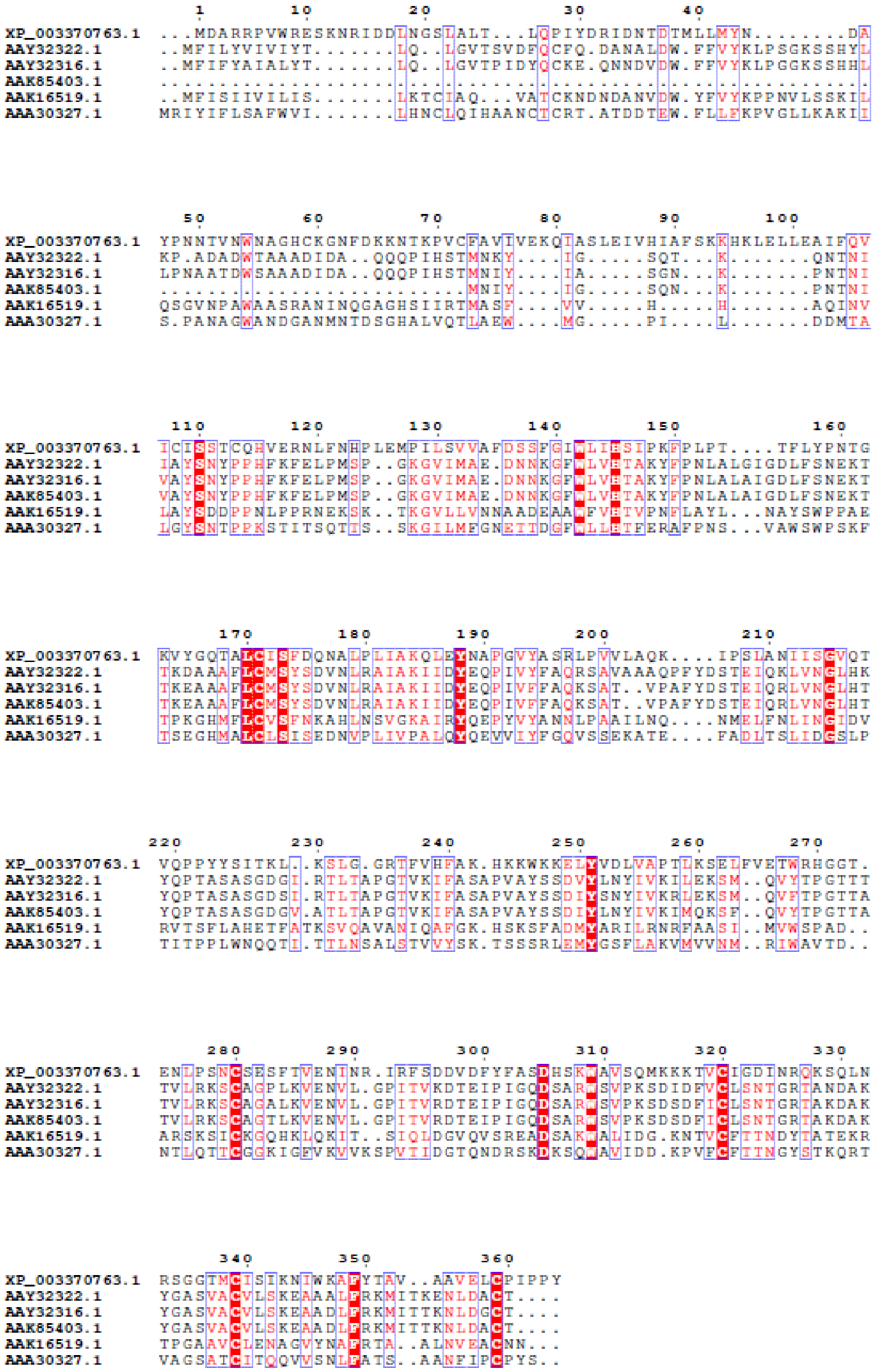

B) Glutathione S-transferases

```
                    1        10        20        30        40        50        60
XP_003371755.1      MAPLYKLSYFDVRGLSEPIRLLFHDQKIEFIDHRFDRNEWPKIKPTIGMFGQVPCLYENG
XP_003373651.1      ...........................................MTNTSKKGQLPFVELNG

                             70        80        90                         100
XP_003371755.1      NPIVQSGAIMRHLGRRFDLYGNADEMTYVD...................EIYEGICDLKR
XP_003373651.1      KEIADSNFIIDELSVHFKKEDCEDKTALEKATLRAFESLIEDSLSWVVVVIRSNHCDVLF

                                                          110       120       130
XP_003371755.1      KYAPFI.................YT.............EHSEEEVEKFTKEVLSVELQKF
XP_003373651.1      NDNSFIRLLPAAFRPIRAIAAKIFSNKLKTKAFHQGIGRHSLTEVTTIGQNL....VKAI

                            140       150       160                 170       180
XP_003371755.1      ENLLKGKKYILNDKISFADYSLFDMLDTLL..........MLSPACLSSFPTLKVYHADF
XP_003373651.1      SVFLGDKTYLSGEKPTKLDAVAFGHLGQLWYTPVESDLKKFIESECANIVQYLERMKTSF

                            190                 200
XP_003371755.1      ITDHPNLKAYL.......KSDDHKK..IRLN.............................
XP_003373651.1      WPDWAQLCVNQSVKVAKEKASDGSKGNAECNAAAELTDKIEAMENGAEEKCDNVSQEEEN

XP_003371755.1      ..........................
XP_003373651.1      AVANSNATTPVAMATENAQPVTIESQ
```

C) Serine proteases

```
                          1        10        20        30        40
ABY60762.1      ..........MILFKCLFLLAYTTLAFANYECGTL..PSTFPKSVRGNRISGGSVATPNS
AAR36900.1      MIRRLFQYTSMTFAWILLFLSAASPSLGAFECGVPHFKPYIWK...SGRIVGGTDVRPHS
ACA28930.1      .MKR.CHYLEIFFYHALLIFCIIIKKTFSEECGKPYFEPYLTNPRNPNRIVGGWVAKPYS
ACA28931.1      .MKR.WHPFGIPFHNAFLLFCIIIKETFSQYCGNPYFEPYLTNPHYPDQTVGEWVARPYS
ACA28932.1      .MKR.WHPFGIPFHNAFLLFCIIIKETFSQYCGNPYFEPYLTNPHYPNQIVGEWVARPYS
AAK31787.1      .MKR.WHPFGIPFHNAFLLFCIIIKETFSQYCGNPYFEPYLTNPHYPNQIVGEWVARPYS

                 50        60        70        80        90       100
ABY60762.1      IPYQARLVFRKAGNRIKLCGGSLVELKP.GNGSQWVLTAAHCAYYAEYGRRFTPDEVQVI
AAR36900.1      HPWQIQLLKSETGGYSSLCGGSLVHFGKPSNGTRFVLTAAHCITTSNM..YPRTSRFTVV
ACA28930.1      FPWTVHILTHVSGLLYESCGGSLISLDS.TNASDTILTASHCVRVNNR..LVNANAITVA
ACA28931.1      FPWTVHVLAHISGFWYESCGGSLISFDY.SNASDTVLTSSHCVRVNNR..LVDANAITVT
ACA28932.1      FPWTVHVLAHISGFWYESCGGSLISFDY.TNASDTVLTSSHCVRVNNR..LVDANAITVT
AAK31787.1      FPWTVHVLAHISGFWYESCGGSLISFDY.TNASTTVLTSSHCVRVNNR..LVDANAITVT

                110       120       130       140       150       160
ABY60762.1      LGAHRPNEEERTQLKVDTKRIISHPRYDDVTVVYDLSLVMLKEYVIYNNEIRSACLPKPD
AAR36900.1      TGAHNIKMHEK.EKKRIPITSYYVQHWNPVMTTNDIALLRLAETVYYNEYTRPVCLPEPN
ACA28930.1      AGVFNIKKLNEPHRVTSKVLAYVSDNFDDVSMENDIAVLRLKVEIQHSEYISPVCLPKQN
ACA28931.1      AGAFDIRDLNEPHRVTSKVLAYMSDNFGIVNKPNDVAMLRLKVKIPHSEYISPVCLPYSY
ACA28932.1      AGAFNIRELNEPHRVTSKVLAYMSDNFGDVGKPNDVAMLRLKVKIPHSHYISSVCLPYPF
AAK31787.1      AGAFNIRELNEPHRVTSKVLAYMSDNFGDVGKPNDVAMLRLKVKIPHSHYISSVCLPYPF
```

```
            170           180           190           200           210           220
ABY60762.1  EPVPLDVPCFASGWGITYQGCRGSDVLRIAEMKPLPKDECRIKPEEHAIVFCAYGVD.RS
AAR36900.1  EELTPGDICVVTGWGDTTENGTTSNTLKQVGVKIMKKGTCANVRSE.VITFCAGAMEGGK
ACA28930.1  QKLPWGKMCFVSGWGLTRESGKPSSKLRQVGIPILRNSNCPFIDAD..DMFCAGDMSGGK
ACA28931.1  QDIPWGETCFVSGWDLSK..GKPSSKLYQVGIPILQKNNCRFVDAH..DIFCVGDVIGGI
ACA28932.1  QEIPYGETCFLSGWGFTR..GRPLSELRQVGIPILRSSNCRFTDAY..DIFCAGDMGEGN
AAK31787.1  QEIPYGETCFLSGWGFTR..GRPLSELRQVGIPILRSSDCRFTDAY..DIFCAGDMGEGN

              230           240           250           260           270           280
ABY60762.1  ATCQGDSGGPVVCLKNNKATLYGIVSYGPPTCGDARHSVKFAKVPAMMQWIQDTARSIDS
AAR36900.1  DSCQGDSGGPLICKKNGKSVQFGVVSYGTG.CARKGYPGVYAKVPSYVTWLNKAAKELEN
ACA28930.1  DSCQGDSGGPLVCKLNGTYVQMGIVSFGDG.CARKDHPGIYTKVPHYVKWIYNQAAKLPD
ACA28931.1  DPSQVDSGGPLVCKLNDSYVQMGIVSFRYG.HAGKHHVGIYSNVPYYSNWIYNQLWWLPD
ACA28932.1  YSFQIDSGGPLVCKLNDSYVQIGIVSFGYN.HAGKHHPGIYSKVPYYLNWIYNQLSWLPD
AAK31787.1  YSFQIDSGGPLVCKLNDSYVQIGIVSFGYN.HAGKHHPGIYSKVPYYLNWIYNQLSWLPD

              290           300           310           320           330           340
ABY60762.1  TVPVVSRFEPKPSEQKVDQKDTQPSPSVSPGRVDVGKKDIMKDLPEIPTRENVKDTPGDY
AAR36900.1  SPEGTVKWASKED........SPVDLSTAS.......................RPT.NPY
ACA28930.1  SSTSSEIGEEKPD........YSNDFHHSWGS..VGNYF...HFPFFDSSDDIWNN.ENF
ACA28931.1  SSNSSYVGVEDDG........IMGLELLHAG.............................
ACA28932.1  SFNSSDIGGEESD........CPDDCYHPWRS..VFKHF...KHRKAS....FRNR.PPY
AAK31787.1  SFNSSDIGGEESD........CPDDCYHPWRS..VFKHF...KHRKAS....FRNR.PTY

              350             360           370
ABY60762.1  KGSDVFN..F...IYRDGFPSHSKFPDLEQIMSDFMS.......................
AAR36900.1  TGSRPT...SPSSGSRPTYPSS.........GSRPTSPSSGSRPTYPSSGSRPTY.....
ACA28930.1  GNENSPFLQLPWSFLSEPMP.......LFRSRLSFINKEAGDWPPYSTNQHFQTY.GRPH
ACA28931.1  ............................................................
ACA28932.1  SH....SLRLTMNENRPPPPPDSQN..FDMESLESTEGDPSDWSPYSTNQHYQSNYDGSQ
AAK31787.1  SH....SLRLAMNENRPPPPPDSQN..FDMESLESTEGDPSDWSPYSTNQHYQSNYDGSQ

           380           390                                   400           410
ABY60762.1  PGSGGMSGGSFFNSP...........RVFIET...........KTINRLPSVSRS.....
AAR36900.1  PSSGSRPTYPYTGSRPTP......QKPV.FPSYQ............KYPPAVQKYIDSLP
ACA28930.1  IGEEGQPQYPLENWPDMNGKYPLHHHSEFHPPYLYSNRPSMNEGHLSRPPNFENP.DEI.
ACA28931.1  ............................................................
ACA28932.1  TGKGNR........................PPYSHSHRPTMNENRPPPPPDSQNF.DSF.
AAK31787.1  TGKGNR........................PTYSHSHRPTMNENRPPPPPDSQNF.DSF.

                                   420
ABY60762.1  .....R..SDVGRFGFNDPSFTFFR.
AAR36900.1  SGTQGTLEYTVTQNGVTTTTYYHFSK
ACA28930.1  ......LKITL...............
ACA28931.1  ..........................
ACA28932.1  ......Y...................
AAK31787.1  ......Y...................
```

D) Chymotrypsin like proteases

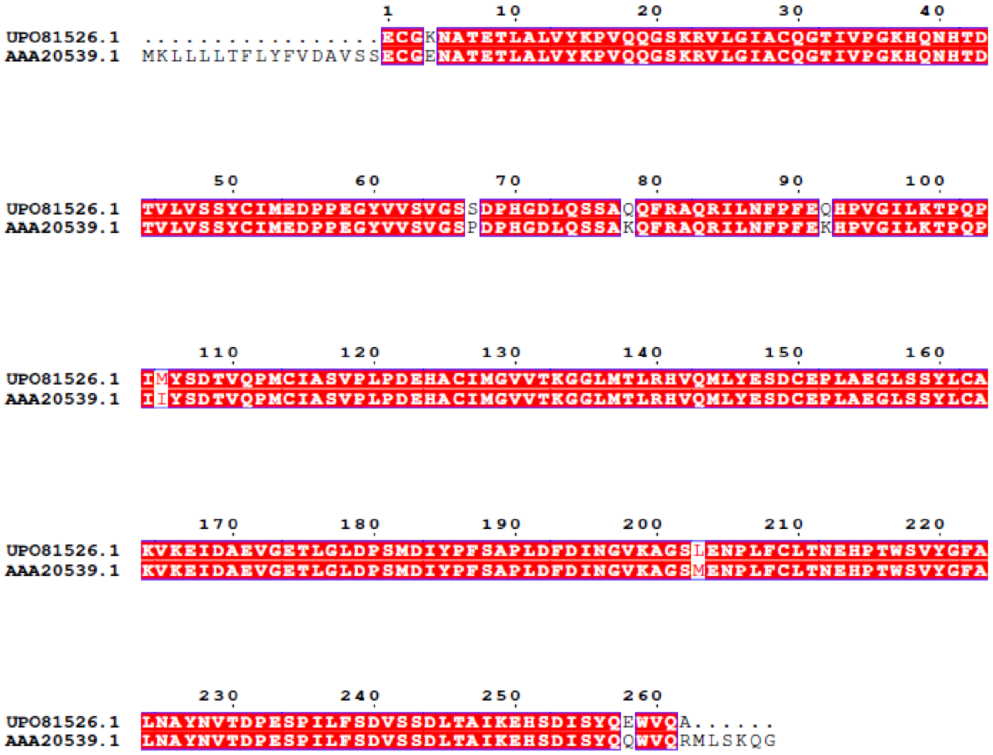

E) Trypsins

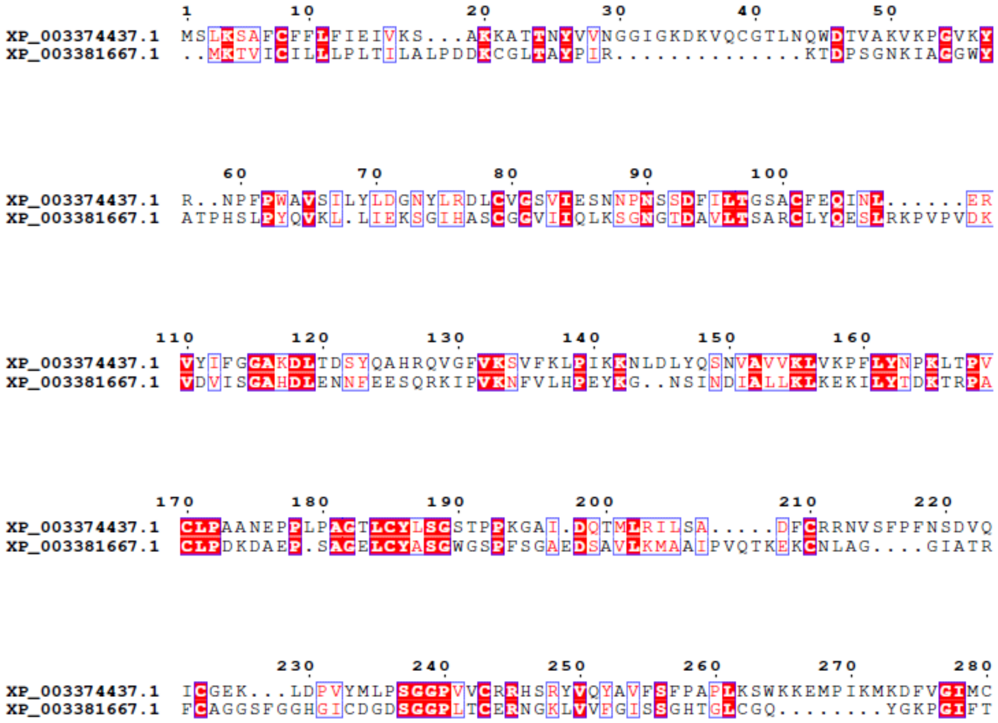

```
                       290
XP_003374437.1  TVSNHLQFIKNSIRVA............................................
XP_003381667.1  KVSSFLDWIKKTDTELDTAVDMSKGKKAENIAQPLDSDETTDAKQAGKVKEQFPCGLSAF

XP_003374437.1  ............................................................
XP_003381667.1  PMKKEQSPSNRVSGGWETRPNSLPYQVKLINQKQGKEFACGGTLIQFKPGNGTFWILTAA

XP_003374437.1  ............................................................
XP_003381667.1  HCIYDNLRKKTLDPEKVQVLVGAHNVYRDSEENRKQIAVQNVIMQPGYNDRTIANDIALL

XP_003374437.1  ............................................................
XP_003381667.1  QLQEPVFYTTVTRPACLPNPGEKPLPTTSCWVSGWGAESSYGEPTAILKVAKVAIWNDAD

XP_003374437.1  ............................................................
XP_003381667.1  CKVDATSSICLGGKADRRGSCQGDSGGPLLCEHNKRMVVFGVSSSVVGHCGQLNQPSIYT

XP_003374437.1  ............................................................
XP_003381667.1  RVTHYLDWLKETSEKAGDLKVTAASSTGGSSATSKPNKAKDDFTVRPSSPAKSVSSVPSQ

XP_003374437.1  .......................................
XP_003381667.1  PAFPGSRFMTPLPTPGRPAASRFYTPPSSGLSRSRFQTL
```

F) Cathepsins

```
                                                                          1
XP_003378245.1  ..........................................................MVS
KRY31298.1      ..................................MQVQLYLTAT.VTVNKQMNRS..LLLS
XP_003372938.1  .............................................................
XP_003379334.1  ........................................MN....TLNRILAAIFALILS
AGR34128.1      .............................................................
XP_003377082.1  MLILLACLEEALCNALVRSSTGILTSEKMNIYPNQSVQFYLNNLTPEINGPIANGLALFVI
ARG41672.1      .............................................................
XP_003379650.1  .............................................................

                         10         20                            30
XP_003378245.1  VKCTVFLFCL...FYCTWAL....PMKQKR..................PLFTNVNHLE...
KRY31298.1      VAVIAFLFLS...IV.TI.......VRDMG..................MMIPNIEQLN...
XP_003372938.1  .............................................................
XP_003379334.1  TEADIPVSCYFEDVAGTW.KFQESGYSTKGPATCENATMDFSRQNIIQLLYPNVALDKFGN
AGR34128.1      .............................................................
XP_003377082.1  SRIAALVGCRSGKIEAAFIYWQRSGLVTGGPYGEKACCLPYSISPCT.MCRPYMLAPK...
ARG41672.1      .............................................................
XP_003379650.1  .............................................................
```

```
                                   40         50
XP_003378245.1  ..R........YMDSKFDKNLLLK.....................................
KRY31298.1      ..WKQHIDAVPFIVLAKAKKIYLR.....................................
XP_003372938.1  .............................................................
XP_003379334.1  RGKWTLIYNQGFEVIVNNRKYFAFFKWIKRDNKFI..........................
AGR34128.1      ...................................................MFLQV.....
XP_003377082.1  ...CQRTCQASYNLSLKRDKYYGKSHYYVNQDEFDIMQEIYQRGPVVAGFKVYHDFLYYIS
ARG41672.1      .......................................................MKILLL
XP_003379650.1  ...........................................MPHSAAPSLFHLFYILLF

                                        60         70         80         90
XP_003378245.1  ..L...LPEM....NAKE......ARSWENFKQFMVEFNKWYETEKLTAEKYNIF......
KRY31298.1      ..FVKGYPPL....TTEQKEMYFKESEWIIYKEI...YGKTYANESEENYRREVF......
XP_003372938.1  ...................................MR...TSSTLLL..LFFNLF....FH
XP_003379334.1  ..SICGK.......TLPGWQHDILGRHWSCFVGTKLHPSIFQATATT..LPMNASQINQQH
AGR34128.1      .......................................VTFVALIK..FSF.......CG
XP_003377082.1  GQFICGNKRCEEEENLTSWEVNFAYV..........EEQEKKNALVK..LNL.........
ARG41672.1      S.LFA...................................F...ALA..ENY.......EE
XP_003379650.1  S.LPC...................................FYSTVFG..IPF.......GS

                           100        110        120                130
XP_003378245.1  .KSNMVIAKRLQEE.EQGTAIYG..P.TIFADMTPEEFRKTH.........LNFNPNNVKK
KRY31298.1      .YANRLKVIRHNEQFDGGAKSYSMKL.NKYSDLTHGEFVQLMNGFKIASKSGDYRPSSVFK
XP_003372938.1  YTAPTKVEVKNAFTKNNVRHGHRSCYRPIFSNEP...................N.MVKTRL
XP_003379334.1  FKHEYYKMADFVSTINSIQTNWTAKIYDEQFYLNRVESLIRANA.YQYHRTAKK.IKSAPI
AGR34128.1      YYEDNYI...QLIKNNQMPKTWKMGLNPYFSGMSKEEILIRMGT.KLMNSSTEF.DSKLS.
XP_003377082.1  .KRRKGT...KLKQKLCMQKEKIMGLNPYFSGMSKEEILIRMGT.KLMNSSTEF.DSKLS.
ARG41672.1      NYERLKVELERQRQANGNTFSWKFGRNAYFKNKSIGEIKKLLGY.RMLPKTVKE.RNEMPM
XP_003379650.1  RNQRLYFN.KMATYINNLQTTWKAGRNPYFETVPSHVIQGMMGV.RRSSK..LE.TNSIPL

                140       150              160          170       180       190
XP_003378245.1  PKRMANIPKSNISERMDWRKFN.....AVTSVK...DQGNCGSCWAFCTVANIEGAWAVKT
KRY31298.1      PL....LFTGDLPLNVDWRSEG.....MVTPVK...DQGHCGSCWAFSATGALEGQNKRKT
XP_003372938.1  RPHEYPGVVENLPKELNWCNYN..GINFCSPTRNQHIPQYCGSCWAMGATSALADRINIRR
XP_003379334.1  T.TEQKLSAVILPEKFDWRNNN..GNNFIGDVR...DQKNCGSCYAFASASMLEARYHILT
AGR34128.1      ..NNNEALIKKLPKHFDSREKWPECEW.IRFIR...DQSNCGSCWAVSAASVMTDRHCIAS
XP_003377082.1  ..NNNEALIKKLPKHFDSREKWPECEW.IRFIR...DQSNCGSCWAVSAASVMTDRHCIAS
ARG41672.1      PEDLLNLENFNYPVEFDSRKHWPQCEKVISFIK...DQANCGSCWAVSSASVMSDRTCIAT
XP_003379650.1  PVISYEHIDMEIPVEFDSRKQWPYCPT.IGEIR...DQSNCGSCWAFGAVEAISDRICIAT

                         200         210        220        230
XP_003378245.1  A..Q.LISLSEQQLVDCDRL..DDGCEGGLPVNAYLEIIRLGGLEK.......EEDYKYTA
KRY31298.1      G..K.LVSLSEQNLIDCSRKYGNKGCSGGLMDNAFEYVKENHGIDT.......EESYPYEA
XP_003372938.1  KGQWPMAYLSVQHVIDCGN...AGSCHGGNHLPVY.AFAHKHGIVD.......ESCNNYQA
XP_003379334.1  QNRERV.TFSPQDVVNC.SPY.SQGCDGGFSYLIAGKYAEDYGMVS.......ERCVAYTG
AGR34128.1      KGQETP.YISDEQILAC.ASS.SYGCSGGMIPSPF.HYWKKMGIATGGPYGDKSCCQPYSI
XP_003377082.1  KGQETP.YISDEQILAC...........GMIPSPF.NYWKKMGIATGGPYGDKSCCQPYSI
ARG41672.1      DGQFTT.LLSDAELLSCCTSC.GYGCNGGYPQRTF.KYWVYSGMPTGGPYGSNDTCKPYPI
XP_003379650.1  DGRQKP.HISSTDLLSCCKIC.GFGCQGGDPHQAW.SFWVKYGLVTGGNYTTHDGCRPYPF

             240        250                               260       270
XP_003378245.1  RSG.KCKFNHTK..........S................AVYINDT.VVLPEDEDAIARYV
KRY31298.1      ADK.KCRFKNST..........I................GATDKGFVDIEPGNETYLMHAV
XP_003372938.1  KDGVCDKFNECGNCV.............TFGQCYPVTNYTLYKVG.DYGPLSGRVEMMAEI
XP_003379334.1  KQ.QQ.............CRTPS...TCERYYA....TDYEYIGG..YYGASNEILMMQAL
AGR34128.1      APCSKCSYT.........ASTPSCKYDCQADYDIPISDDKFYASE.HYHVSSNQYEIMNEI
XP_003377082.1  APCSKCSYT.........ASTPSCKYDCQADYDIPISDDKFYASE.HYHVSSNQYEIMNEI
ARG41672.1      PPCSNCSE..........TRTPKCSKSCISTYPLSLNEDRHYGST.YYQFWLGEKSMMKDI
XP_003379650.1  APCNHHSNGTYGPCSHDLEPTPVCKKACQSTYKIQYNKDKYYGLK.AYSLHNKASDLQKEL

                      280         290       300            310       320
XP_003378245.1  SENGPVAVGLNAD..AMMFYRSGIAHPSRLMCSP.....DGINHGVTIVGYDVKESLFWST
KRY31298.1      ATIGPLSVAIDASHESFQFYSSGVY..FEPMCSS.....QFLDHGVLVVGYGSL....KGK
XP_003372938.1  YKNGPIACGIAV.TDSFEAYTGGIYAEHKLL........PIVNHII...............
XP_003379334.1  VKNGPIAVGFEV.HDDFLSYSHGIYHYTSAVSPLKWNPFVEVNHAVIIVGYGTDE..MTKE
AGR34128.1      YTHGPVVAGFIV.YEDFTYYISGIYQQTTYV........AMGGHAIRIIGWGEE....NGI
XP_003377082.1  YTHGPVVAGFIV.YEDFTYYISGIYQQTTYV........AMGGHAIRIIGWGEE....NGI
ARG41672.1      SLYGPIVAGMSV.YEDFLHYKEGVYTQESGM........FLGGHAVRIIGWGEQ....DNI
XP_003379650.1  MMNGPMEVAFEV.YEDFLLYKTGVYQHHTGS........VLGGHAVRLLGWGEE....NGV
```

```
XP_003378245.1  PYWIIKNSWGPNWGEKGYYYLYRGK.GVC........GIDQMASSVVID.....
KRY31298.1      DYWIVKNSWGTSWGNDGYIFMARNKNNSC........GIASFASYPII......
XP_003372938.1  ..........SVGEHGFFRIVTSE.FKNNTGRHYNLAIETMCAYADPVTF...
XP_003379334.1  KYWIVKNSWGRKFGEDGYFRIRRGT.NEC........GIESLAFQATPIIL...
AGR34128.1      PYWLIANSWNTTFGEKGFFRIRRGT.NEC........RIESEVYTGIPKLRLTL
XP_003377082.1  PYWLIANSWNTTFGEKGFFRIRRGT.NEC........RIESEVYTGIPKLRLTL
ARG41672.1      PYWLVANSWNTTFGEDGLFKIRRGF.DEC........GIESYVSAGRAKV....
XP_003379650.1  PYWLLANSWNTEWGDKGFFKIYRGR.NEC........GIESEAVAGLYKKPT..
```

G) Aminopeptidases

```
EFV57850.1      MAKRQTTRLLEKLRAIMKSKVYAMH.EISAYIISSNDAHFSEYTADCDRRIAFISGFTGS.
EFV57052.1      MSRK..........GLM.LGIYSSESKISV..EEQLTCAAKKFNADNAGKLLTYLNYTEPL
XP_003377703.1  MSRK..........GLM.LGIYSSESKISV..EEQLTCAAKKFNADNAGKLLTYLNYTEPL

EFV57850.1      .....RGTAVITDKQAALWTVGIYHLQASKELGDDWILMKE......GLPETP........
EFV57052.1      KEGKCRMFYGISDKFDALAVVGIGK......QGEEYVEEEDLHQGRENVRRAVAIGTVALR
XP_003377703.1  KEGKCRMFYGISDKFDALAVVGIGK......QGEEYVEEEDLHQGRENVRRAVAIGTVALR

EFV57850.1      EIEQWLANVLPAGSFVGVDP......FLLTEEAFTRCKKKLS.......DHKIELKEVATN
EFV57052.1      DVGMREIYIDPCGCADGEYASAAAEGAFLSTFNFDELKSKPDSKKQNPTLHLYDYGGTGSV
XP_003377703.1  DVGMREIYIDPCGCADGEYASAAAEGAFLSTFNFDELKSKPDSKKQNPTLHLYDYGGTGSV

EFV57850.1      LVDIVWGEDRPLRTGGMVYF..........L..................PTFHTGRSWEQK
EFV57052.1      ELEKAWNRGQKLAEGENVVRRLSDMPANMLTPTLFADYAARVLADYSNIKVIKRDRKWAEE
XP_003377703.1  ELEKAWNRGQKLAEGENVVRRLSDMPANMLTPTLFADYAARVLADYSNIKVIKRDRKWAEE

EFV57850.1      ISDVCSIMAKNRVQHLVLSALDEIAWLLNLRGSDIPYNPVFFAYVVISNEDA..VSLFIDE
EFV57052.1      KK...................MGAFLAVAQGSDQP..PVELELHLSAKKSTKPPLCFVGK
XP_003377703.1  KK...................MGAFLAVAQGSDQP..PVELELHLSAKKSTKPPLCFVGK

EFV57850.1      GKIAKTILDKFLMNNSSLRVNCFHYDAISDYMTNCLVDKDDSSLRIWLPQGTSHALCSLVP
EFV57052.1      ...................GVCFDSGGISLKP.............................
XP_003377703.1  ...................GVCFDSGGISLKP.............................

EFV57850.1      ESNRYTAQSPILLLKAVKNKSEVRGMRNAHIKDAVAHCMFFGWLEKQIMFFKNQEITELDA
EFV57052.1      ................S......KSMGSMRFDMTGAACVLSTIL..........TLAKLGS
XP_003377703.1  ................S......KSMGSMRFDMTGAACVLSTIL..........TLAKLGS

EFV57850.1      SAKFEQFRSMQHDYKGPSFKTISAFGSNASIIHYSPSEQSNRLLNDKNLYLIDSGGQYIDG
EFV57052.1      ELPFDVIGLIP..................LVENLPSGK..AIKPGDVVYAMNGMSIEVEN
XP_003377703.1  ELPFDVIGLIP..................LVENLPSGK..AIKPGDVVYAMNGMSIEVEN
```

```
                      430       440       450       460            470
EFV57850.1        TTDTTRTFMFSECTEHQRRCYTMVLKGHIALARMVFPEGCI....GARI..DALSRTFLWK
EFV57052.1        T.DAEGRLIL.....ADGLCYADTFKPSHVIDIATL.TGAILVALGDSAAGVFTNDKALWK
XP_003377703.1    T.DAEGRLIL.....ADGLCYADTFKPSHVIDIATL.TGAILVALGDSAAGVFTNDKALWK
```

```
                 480       490                        500                   510
EFV57850.1        QGLDYPHGTGHGI................GHHLCVHEGPSGFG..P............SLG
EFV57052.1        KIHKAAGNTGERVWRLPLYKHFSEQVKLESIDLCNTSKPSFQGLGGSCIAAAFLKEFTTCN
XP_003377703.1    KIHKAAGNTGERVWRLPLYKHFSEQVKLESIDLCNTSKPSFQGLGGSCIAAAFLKEFTTCN
```

```
                        520       530       540       550       560       570
EFV57850.1        SWNCEGILENMILTIEPGYYENENFGIRIENAYVVVPAETEFNYENKKYLRFEPLTLVPIQ
EFV57052.1        SWMHIDIAGVVERKGSNPLYNTS...............................MTGKPLR
XP_003377703.1    SWMHIDIAGVVERKGSNPLYNTS...............................MTGKPLR
```

```
                       580       590       600       610       620
EFV57850.1        KKMIVREMMTDEEVNLDVAIQWLNEYHAKCLESVSDGLMHSGDVDAVKWLHEETRPF
EFV57052.1        ALVEFCELLAKSS............................................
XP_003377703.1    ALVEFCELLAKSS............................................
```

H) Aspartyl proteases

```
                  1        10        20        30        40        50        60
XP_003380300.1    MLRTICILAAVLVVSCGQIQPVRLHRGQSVLKQLLEKGSYVDYGRKLEQYVHFLRKKYEN
QOQ72494.1        .MRRF.LIATLFLITTGFSAKVPIKEI...............ALRIPREWILSIRKLYDN
```

```
                         70        80        90       100       110
XP_003380300.1    RLHKTPGEIDEILHN.YMDAQYYGEISIGTPPQNFTVIFDTGSSNLWVPSSKC.......
QOQ72494.1        DNSSSY..TESLFDSLKHLSFYYVDVEIGTPAQKFRLLVDTASSSLIVVGTEYDSDIATA
```

```
                                             120       130       140       150
XP_003380300.1    ....................SFFDIACWLHNRYNSKKSSTYEASGETIEIRYGSGSMRGF
QOQ72494.1        CRKQCHDEDHCCIRVCYKNFNASDWNVWRKNRFNADKSETFVKKSTFKEYAFND.FAMGI
```

```
                       160       170       180       190       200       210
XP_003380300.1    KSKDTVCIASLCVKGQGFAEATSQPGLAFIFAHFDGILGMAFPSIAVGGIQPVFQAMIEQ
QOQ72494.1        TGTDSICLGEKCAKHQHFGIAN.TLGLLFFLLPCDGSLGLGFQTLNKDYSVPLMQRLLRQ
```

```
                       220       230        240        250       260       270
XP_003380300.1    NLISEAVFAFWLNRNPED.DLGGLISFGTVDE.KYYIGNITWVPLVNQRYWEFNMETIKV
QOQ72494.1        DKASPPTFTIWIDQSNDDGNFDGQLTIGEYDEEHCQMQTCQWMKLARRRYWEFEFENIVV
```

```
                           280         290       300       310       320
XP_003380300.1    GDEHVGCIDGCTTIA...DTGTSLIAGPKDEVERLQEAIGAKPLIMGQYYVSCNEVDSLP
QOQ72494.1        TNDLEIRNADRTPMKGMFNSRIPWIAMASAHVKAICNELELEFVSYMPIKIPCNRQHSLK
```

```
                  330       340       350       360       370       380
XP_003380300.1    NVQMKIGGRMFDLKPEDYVLRVKQMGQSICLSGFMGLDLPPQVCKLWILCDIFIGLYYTV
QOQ72494.1        PMKLVVENKIIPIPTSAYFAQKND....TCYLMIIA..AETAQCIDVEFCNYFMRQNCHV
```

```
                  390       400
XP_003380300.1    FDVGNSRLGFANATKLHS
QOQ72494.1        YNPHANKMAICNRKQSE.
```

I) Cystatins

```
                                              1        10          20        30
XP_003379766.1    ............................MSNICGGVKEE..REPTEAETAIALGLRSDVE
BQ692489.1        ....MLHDLLLFSVAYFVVCNINCAVVVGEPIVGGLTPADDDDPKVRQMIQYAVQQLNEK
XP_003369399.1    .......MSALAAFIFFFMAVM.......PEI........NADLSELDEAKNYIYQSDLQ
QTG10996.1        ..............................QILGETTHYGRNDPVMLRNAHEALFSSDLK
ABY60755.1        SIQKMSFMHCIFVVLFFAVGE........AQILGETTHYGRNDPVMLRNAHEALFSSDLK
```

```
                            40        50        60        70        80
XP_003379766.1    ..NQLNRKFKHFRPVSIRTQIVAGINYFFKVMVDEDDFIHLRVFKNLQNETQLHGVQHEV
BQ692489.1        SKEPFLRKLISLK..NAAVQVVQGALYHLNILVAETDCEKGE.................N
XP_003369399.1    TGRGNFRKVLKVR..NVD...TSDGLS.LTIDALPTTCPVSS.................E
QTG10996.1        QESGVFHKLLELE..ESS...TMGILTTMKVVMQDTDCPVSF.................V
ABY60755.1        QESGVFHKLLELE..ESS...TMGILTTMKVVMQDTDCPVSF.................A
```

```
                  90        100              110        120
XP_003379766.1    IRFNHYYLQNIS.......KKLCDCFLKKFI.KWNRSVKTS...................
BQ692489.1        VE.......NVQNCKTTPDGLSQECFIKIWEREWLNFIKVMKTKCKEASLPTVNITDTSK
XP_003369399.1    KSLEEVYSDECRITK..DEYDKIECHLKLDQNKS...GQ...IECT..............
QTG10996.1        LLS..YY.DVLVNCQ..GEGRRKHCVMEYTHRNP...SKATVSKCFEEVEEPLIVPQRVK
ABY60755.1        LLS..YY.DVLVNCQ..GEGRRKHCTMEYTHRNP...SKATVSKCFEEVEEPLIIPQRVK
```

```
XP_003379766.1    ............................................................
BQ692489.1        PA............................DDP.................QVGVIAT...
XP_003369399.1    ............................................................
QTG10996.1        MIGGRAVYIDSNADMEEQMQMLGETTHYGRNDPVMLPKAREALFSSDSKEQSGVLHKLVE
ABY60755.1        MIGGRAVYIDSNADVEEQMQMLGETTHYGRNDPVMLPKAREALFSSDSKEQSGVLHKLVE
```

```
                                                            130       140
XP_003379766.1    ...................................VHFAYCLLSTLLKRCVIHLIFPCRS
BQ692489.1        .PENSTIPAEEIITTVA...........................................
XP_003369399.1    .................................YYAV.......................
QTG10996.1        LEESSTM...GILTTMKVVIQDTDCRVSSAYSSYYDVLHYCHGKGRRKYCTLEYRYRTPS
ABY60755.1        LEESSTM...GILTTMKVVIQDTECRVSSAYSSYYDVLHYCHGKGPRKHCTLEYRHRTPS
```

```
                    150
XP_003379766.1    VSELSTF.....................................................
BQ692489.1        ............................................................
XP_003369399.1    ............................................................
QTG10996.1        TATLSECFEEVEEPLIVPQRVQRVNGRTIYIDPTADVEEQIVPQRSQMLGGTTRYNDSNV
ABY60755.1        TATVSECFEEVEEPLIVPQRVQRVNGRTIYLDSSDDVEEQVVSQRSQMLGGTTKYTDSNV
```

```
XP_003379766.1     ............................................................
BQ692489.1         ............................................................
XP_003369399.1     ............................................................
QTG10996.1         RVKEEVKQAIFESDKKKSSGTYLLLDKILDGLNMGISSRFQVLVKETECNIKEKAYDSYK
ABY60755.1         HIKEEVKQAIFESDKKKSSGTYLLLDKIVEGFNMGISSRFQVLVKETECGIKEKAFNSYE

XP_003379766.1     .............................
BQ692489.1         .............................
XP_003369399.1     .............................
QTG10996.1         DVYANCSGSGDSKVCSVEYKYFDPTKSTVEC
ABY60755.1         DVYKNCSGSGDSKVCSVEYKYFDPTKSTVEC
```

J) Serpins

```
                                           1        10        20        30
XP_003379899.1     ........................MLSVIFS......AVAVGLVGWDPKSGRFSNAQLSMD
AAF63473.1         .METEIAKPLADFAYSLYQ..LEEAGNVFFSPVSIFLALAMVFFG...SNGNTNTQLLNVM
ABI32311.1         .METEIAKPLADFAYSLYQ..LEEAGNVFFSPVSIFLALAMVFFG...SNGNTNTQLLNVM
ABY60739.1         .METEIAKPLADFAYSLYQ..LEEAGNVFFSPVSIFLALAMVFFG...SNGNTNTQLLNVM
AEO72145.1         MSSVNFDISMANFAMELYRQSFSNQSNSFFSPYSIVLTLAMTYFG...SSGRTKQQLKDRL
XP_003377380.1     MSSVNFDISMANFAMELYRQSFNNQSNVFFSPCSIVLTLAMTYFG...SSGTTKQQLKDRL

                         40        50        60        70        80
XP_003379899.1     FPLADETDERCLCSKHLQPVCGKHRGQSYVYRNLCTLACNQKHLDGLLYSYDGFCCQE...
AAF63473.1         FKAGWKKNR.T..KKAMRSFVSSLTIDEYYDA........SLKLANRLYANDQYPILHPFL
ABI32311.1         FKAGWKKNR.T..KKAMRSFVSSLTIDEYYDA........SLKLANRLYANDQYPILHPFL
ABY60739.1         FKAGWKKNR.T..KKAMRSFVSSLTIDEYYDA........SLKLANRLYANDQYPILHPFL
AEO72145.1         FSVSDDQLQ.ASLDGIFQSLQGDQHQQEQLTM........QLHLANRLFARNNLKLLPAYL
XP_003377380.1     FS.....................................................LLPAYL

                      90       100       110       120       130
XP_003379899.1     ...RPCAANALPVCDLRGRIYRNQCHFENEQCVVWKKHRQIIKK..........ANVCPCS
AAF63473.1         KDVKRYLSSDLVSVNF.....AD.TEAARLQINKWVSDQTNHKINDLLQSGTVEANTRLIA
ABI32311.1         KDVKRYLSSDLVSVNF.....AD.TEAARLQINKWVSDQTNHKINDLLQSGTVEANTRLIA
ABY60739.1         KDVKRYLSSDLVSVNF.....AD.TEAARLQINKWVSDQTNHKINDLLQSGTVEANTRLIA
AEO72145.1         TRIQKTFKADVDLVDF.....SN.GAAAAEKINRWVANETKDRIKNLIPPDVLDEMTCLVL
XP_003377380.1     ARIRQTFKADVDLMDF.....SN.GAAAAEKINRWVANETNDKIQNLIPLDVLDEMTCLVL

                   140       150       160                             170
XP_003379899.1     KPCPYYEQPVCDSYGKTHKNRCLFKR.......................EQCYLKLAY...
AAF63473.1         VNAIYFKASWDEVFDEAHTKPKKFYPTPHSSIKIPMMTQTNGYSYYETEDYQFLGMDYYPE
ABI32311.1         VNAIYFKASWDEVFDEAHTKRKKFYPTPHSSIKIPMMTQTNGYSYYETEDYQFLGMDYYPE
ABY60739.1         VNAIYFKASWDEVFDEAHTKRKKFYPTPHSSIKIPMMTQTNGYSYYETEDYQFLGMDYYPE
AEO72145.1         VNAIYFKGNWQTRFAPESTSKQYFSVDQNTNKLVDMMHVNDTFRHAEHEQFQILQLPYESS
XP_003377380.1     VNAIYFKGNWQTRFARESTSKQYFSVDQNTNKLVDMMHVNDTFRHAEYEQFQILQLPYENS

                                          180       190       200       210
XP_003379899.1     .............GIEVT.FKQEGPCCVTRCHGRSSQQRDITICDSKGRTHRN........
AAF63473.1         YLKMFILLPKSGKTLSELQQKFNGETLLNLVSKVSGAEVKVTIPKMKFEKQMNLVEALKKL
ABI32311.1         YLKMFILLPKSGKTLSELQQKFNGETLLNLVSKVSGAEVKVTIPKMKFEKQMNLVEALKKL
ABY60739.1         YLKMFILLPKSGKTLSELQQKFNGETLLNLVSKVSGAEVKVTIPKMKFEKQMNLVEALKKL
AEO72145.1         KLAMYVLLPKEKFGLEKLVNQLSGEQLLDSMEAVTSKKVSITFPKFKLEETLPLKKILLQL
XP_003377380.1     KLAMCVLLPKEKFGLEKLASQLSGEQLLDYMEEMISKKVSITFPKFKLEETLPLKKILLQL

                                220       230
XP_003379899.1     .........LCDFDKMWCQERRLGISTARIMHKGSCR........................
AAF63473.1         GIEDLFIPGKADLS.GICVKEKLYVS..DIVHKAYLE................FNEEGTEA
ABI32311.1         GIEDLFIPGKADLS.GICVKEKLYVS..DIVHKAYLE................FNEEGTEA
ABY60739.1         GIEDLFIPGKADLS.GICVKEKLYVS..DIVHKAYLE................FNEEGTEA
AEO72145.1         GLTSMFDHSMADFS.MMTGDRSVIVS..DAFHKAFIE................VNEEGSEA
XP_003377380.1     GLTSMFDHSMADFS.MMTGDRSVIVS..DAFHKAQIEVIVPIVHNTHYTGRLFVNEEGSEA
```

```
                   240       250       260       270       280
XP_003379899.1    NVT........SAKEI..RTPFIAPFPDAFWPALSAD..SSFWSKLINHQQLYPYW
AAF63473.1        AAATAVRIVPMSGVMYEDSFEFVADHPFLFFIFDSRSKAILFIGRFSGN.......
ABI32311.1        AAATADRIVPMSGVMYEDSFEFVADHPFLFFIFDSRSKAILFIGRFSGN.......
ABY60739.1        AAATADRIVPMSGVMYEDSFEFVADHPFLFFIFDSRSKAILFIGRFSGN.......
AEO72145.1        SAATAVVAMLRSAQHVAPPAVFIADHPFMFLIADMQTQTILFMGSYRG........
XP_003377380.1    SAATAVVAMLRSAQHVAPPAVFIADHPFMFLIADMQTQTILFMGSYRG........
```

K) 21 kDa excretory/secretory proteins

```
                                         1       10        20        30
UPO81527.1    .......................AKSLDAVDDKLKRCTEKQTEICAQTECKAEDAIMTDL
AAB48489.1    QNMHCQFILSLVFFSLNTVFFTAGDSLDAINDKLKRCTEKQTEICAQTECKAEDAAMTDL

               40        50        60        70        80        90
UPO81527.1    LLEGESDITDHPDFRLYATCMQRCCARLNGAQVAPLKEEEKRRGPSKLPFQSIFEVADQK
AAB48489.1    LLEGESDIFDHSDFTSYATCMHRCCARLNGAAVPPLKEGEKRRGPSKLPFQSIFEVADQK

              100       110       120       130       140       150
UPO81527.1    TVERCDETMCKSHRKKYETLVALTSNYKKLRSSQELKDYKECIERCDAKLNGLQ
AAB48489.1    TVERCDETMCKSHRQKYENLVARTSSYKKLRSSQELKDYKECIERCDAKLNG..
```

L) 53-kDa proteins

```
              1        10        20        30        40        50        60
AAK29415.1    MFSITLKLLIIAFINFQLCTCSSDTDKVEVEEMVFSVPLFKPQNVPQIKEKKLKKLLEPL
ABD66079.1    MFSITLNLFIIAFVNFQLCTCSTDNENVAIKEMTFSLPISVLQDERQFDGKKLKKLLKPL
AAA97512.1    MFSITLNLFIIAFVNFQLCTCSTDNENVAMKEMTFSVPISVLQNERQFDENKLKKLLKPL

                       70        80        90       100       110       120
AAK29415.1    GKLYKTPSDVGTPISSSEASESVEKMIFELNRLIQAKYSYIFKQYHKLKTVHQAEKCDST
ABD66079.1    GKLYNTSSDEGIPINTNEATLSVEKMMVELNRLIQKEYSFLYKQYQKLKTVLQAEKCDST
AAA97512.1    GKLYKTPSDKGIPISRTEATLSVEKMMVELNRLIQKEYSFLYKQYQKLKTVQQAEKCDDT

                      130       140       150       160       170       180
AAK29415.1    TNVYTITLQKTDCKAEQIIKGSPSTNCSNVEDSHPISCTILSKVTSENETIIGAFCHVNM
ABD66079.1    TNVYTVTLQNTDCESKSIIEGSPATNCSDVEHHHPLSCSILSKVASADEKIIGAYCSVGL
AAA97512.1    TNVYTVTLQNTDCESKPIIEGSPATNCSDVENKHPLSCSILSKVASAEEKIIGAYCSVHL

                      190       200       210       220       230       240
AAK29415.1    QKKLAKKKSICELFRYPGEEKFKTFVPKEVSSLFADAIVYIPQGQRPQPNEKYYKNYRGR
ABD66079.1    EESFPKKKSICKLSRYPGEEKFKTFVPEEVSSWFHDAIVYVPTGNRPLSNSMHSNNYRGR
AAA97512.1    EESFPKKKSICKLSRYPGEEKFKTFVPEDVSSWFHDAIVYVPTGNRPQSNSKHSNNYRGR
```

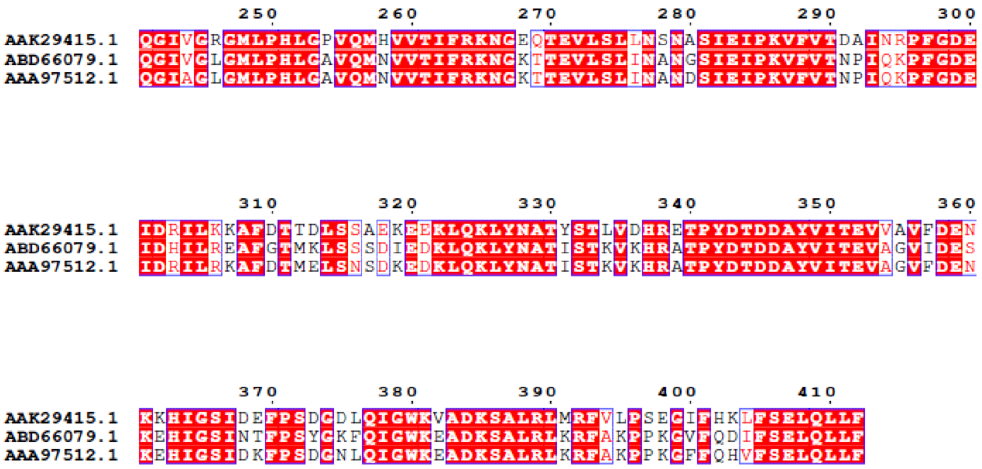

M) Calreticulins

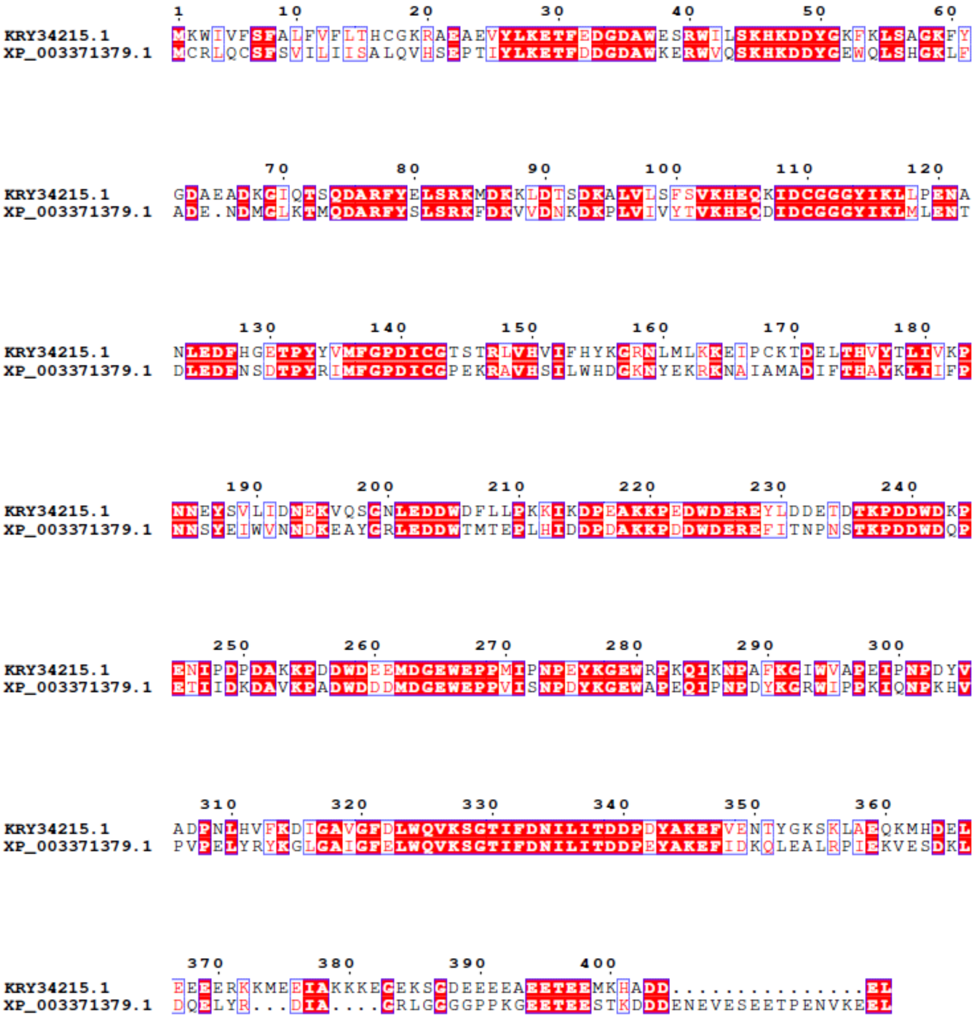

Supplement: Supplementary file 1 — Sequence aligments of cloned Trichinella recombinant proteins with common names. Aligments were made using Clustal Omega and visualized with ESPript 3.0 online software. [file mmc1.pdf]
